# Smooth muscle contractile cytoskeleton in health and disease

**DOI:** 10.1007/s10974-026-09731-4

**Published:** 2026-04-22

**Authors:** Glenn Carrington, Malika Zahedi, Michelle Peckham

**Affiliations:** School of Molecular and Cellular Biology, Faculty of Biological Sciences, University of Leeds, Leeds, UK

**Keywords:** Smooth Muscle, Cytoskeleton, Actin, Myosin, Caldesmon, Calponin

## Abstract

Smooth muscle cells are widespread in the human body, found in a range of tissues from the vasculature to the gut, airways, bladder and so on. They can be found as isolated cells or organised into syncytia (groups of cells). The organisation of the contractile cytoskeleton (smooth muscle actin and myosin filaments) is less well understood than that of striated (cardiac and skeletal) muscle. This review aims to explore what is known, the potential effects of mutations in smooth muscle actin, myosin and tropomyosin and how they lead to disease, and to highlight what we have still to learn about the smooth muscle contractile cytoskeleton.

**Supplementary Information:**

The online version contains supplementary material available at 10.1007/s10974-026-09731-4.

## Introduction

### Smooth muscle

Smooth muscle is widely distributed in the body. Its behaviour and organisation depend on its function. Smooth muscle cells can either be found as individual cells, or in groups of tightly connected cells known as syncytia (Peckham 2011). Their contractile behaviour can be either be tonic (slow and continuous), or phasic (fast contraction and relaxation). For example, individual smooth muscle cells in the vasculature are organised into rings around the blood vessels, their contractile behaviour is almost always tonic and they regulate vascular tone and thus blood pressure (Fisher 2017), with the exception of smooth muscle in the hepatic portal vein, which is phasic (Fisher 2010). Rings of individual smooth muscle cells found in sphincters, which open and close to regulate flow, are also tonic. Elsewhere, organised syncytia of smooth muscle cells, found in the gastrointestinal (GI) tract, stomach and bladder, contain a mix of tonic and phasic muscle. For example, smooth muscle in the proximal part of the stomach is tonic, while in the distal region is phasic (Fiorenza et al. 1987). Likewise, in the tubular small intestine, smooth muscle cells organised into syncytia can also show tonic or phasic contraction. Individual smooth muscle cells, found in the airways and in glandular ducts (e.g. salivary glands, sweat glands, and glands in the reproductive system) as myoepithelial cells, regulate the diameter of these structures and are phasic (Peckham 2011).

Smooth muscle cells are mononucleated, spindle shaped cells that have a highly plastic cytoskeleton. They can modify the organisation of their contractile cytoskeleton, dedifferentiate, enlarge (hypertrophy), or alter their resting tone (level of partial contraction at rest) and contractile ability in response to external signals (reviewed in (Beamish et al. 2010). For example, in the terminal region of the small intestine (ileum) and in the large intestine, an increase in size and number of smooth muscle cells in Crohn’s disease results in restriction (contraction) (reviewed in (Veisman et al. 2024). Dedifferentiation of vascular smooth muscle cells (VSMCs) in the vasculature is strongly linked to atherosclerosis (Naiya et al. 2024). The dedifferentiated cells are also termed synthetic or non-contractile, and in addition to remodelling their cytoskeleton, proliferation and migration increase, and they lose their spindle shape (Cao et al. 2022; Thakar et al. 2009). The VMSCs not only dedifferentiate but transform into cell types such as macrophage-like cells, and eventually foam cells, contributing to the formation of plaques in this disease (Allahverdian et al. 2014; Chappell et al. 2016). An increase in the numbers of smooth muscle cells in small airways can remodel and restrict airways in chronic obstructive pulmonary disease (COPD) (Chung 2005), and in asthma, this also includes smooth muscle cells in the larger airways (Hough et al. 2020). The developmental origin of smooth muscle cells is diverse (reviewed in (Donadon and Santoro 2021).

### Mechanosensitive behaviour, plasticity and contractile protein expression levels

Smooth muscle cells contain lower amounts of muscle myosin, and its turnover rate is higher than that of muscle myosin in striated muscle. The myosin content of smooth muscle is estimated to be about 5 times lower than that of skeletal muscle (Murphy et al. 1974) or less than 20% of the total protein content (Bagby 1986) and the half-life for smooth muscle myosin (reported for oesophageal smooth muscle) is ~ 5 days (Lewis et al. 1984). In skeletal muscle, over 40% of the myofibril mass or over 50% of the total protein content is made up of myosin (Murphy et al. 1974; Yates and Greaser 1983). The half-life for myosin in skeletal muscle is approximately 12 to 17 days in mice (Rolfs et al. 2021) and between 4 and 14 days in the heart (Gugel et al. 2025).

The organisation of smooth muscle cells around lumens (gut, vascular, etc.) constantly exposes them to stretch and release. Thus, it is not surprising that expression and organisation of the contractile cytoskeleton depend on sensing mechanical forces (Mercado-Perez and Beyder 2022). Whether organised into syncytia, or present as single cells, smooth muscle cells can sense how much they are stretched through several different mechanisms. One of these is through the activation of mechanosensitive ion channels such as Piezo1, which increases levels of cytosolic Ca^2+^ (reviewed in (Bautista et al. 2026; Davis et al. 2023). Another is through the Hippo Pathway (reviewed in (Albinsson et al. 2025)).

The Hippo pathway is activated in smooth muscle cells by low mechanical tension (a stretch of a few percent - reviewed in (Daoud et al. 2022; Totaro et al. 2018)). Activation of the canonical pathway (multiple kinases (MST1/2 (mammalian STE20-like kinase 1/2), SAV1 (protein Salvador homologue 1), MOB1A/B (MOBKL1A/B) and LATS1/2 (large tumour suppressor kinase 1/2) leads to phosphorylation of two downstream transcription regulators, YAP (Yes-associated protein 1), and TAZ (WW-domain-containing transcription regulator 1). Once phosphorylated, YAP and TAZ are blocked from entry into the nucleus, preventing their interaction with one of the four TEADs (transcriptional enhanced associated domains). Instead, they are degraded by the ubiquitin ligase pathway, and cell growth is blocked (reviewed in (Currey et al. 2021; Fu et al. 2022)). Myocardin, a transcriptional co-activator with serum response factor (SRF), is a downstream target for YAP/TAZ-TEAD signalling, and regulates expression of smooth muscle myosin heavy chain, actin and myosin light chain kinase. YAP and TAZ phosphorylation also blocks their interaction with myocardin, leading to decreased expression of smooth muscle specific proteins (Arevalo Martinez et al. 2023). More recently, the non-canonical Hippo pathway was also found to be important. MST1/2, normally inhibits proliferation and promotes apoptosis. However, in pulmonary vascular cells, it works in the opposite way, by interacting with proteins that regulate mitosis BUB3 (Budding Uninhibited By Benzimidazoles 3 Homolog), and/or CDC20 (cell division cycle protein 20), to drive proliferation and survival of pulmonary arterial smooth muscle cells (Dey et al. 2025; Kudryashova et al. 2022).

### Model systems for investigation of smooth muscle cells

Progress in understanding the smooth muscle cell contractile (muscle actin and myosin) cytoskeleton has been slower compared to striated muscle. This is partly because there is a lack of suitable smooth muscle cell lines that can be used to investigate the cytoskeleton, requiring the use of primary cultures. Isolated smooth muscle cells rapidly dedifferentiate when isolated and cultured and so cannot be easily transformed into a cell line (Chamley-Campbell et al. 1979). This is likely because they are not exposed to the same mechanical cues that they experience in vivo, and effects on downstream signalling pathways, such as the Hippo pathway, gradually switch off expression of smooth muscle specific genes.

Many groups isolate and culture primary human venous smooth muscle cells. These cells are isolated from the saphenous vein, which is commonly used in heart surgery to repair and revascularize atherosclerotic coronary arteries (Turner et al. 2007), subject to ethical approval. Once the inside (endothelium or intima) and outside (adventitia) layers have been removed from the vessel, the middle layer (media) is mainly comprised of smooth muscle cells, and few if any fibroblasts. Plating primary VMSCs onto laminin and adding IGF-I (insulin-like growth factor-I) to the medium may help prolong the differentiated phenotype for primary cells (Hayashi et al. 1998). A recent method to isolate vascular smooth muscle cells from the skin may also prove to be useful (Hussain et al. 2025). Primary vascular smooth muscle cells can also be isolated from aortas and purchased commercially.

It is important to use primary smooth muscle cells at early passages and ensure that they have not dedifferentiated. One way to do this is to quantify the expression of smooth muscle specific markers (SMM heavy chain - encoded by the gene *MYH11*), calponin, smooth muscle tropomyosin isoforms, α and/or γ smooth muscle actin, Transgelin (SM22), and smoothelin (Beamish et al. 2010; Milton et al. 2011). Interestingly, the smooth muscle ‘proteome’ (https://www.proteinatlas.org/humanproteome/tissue/smooth+muscle) reported by the human protein atlas, reports just 50 upregulated genes, and surprisingly *MYH11* is not included.

Immortalised smooth muscle cell lines have also been used in some studies (e.g. rat A10 and A7r5 lines along with human IM-HASMCs). Rat 10 cells were reported to show a dedifferentiated phenotype (Rao et al. 1997), expressing non-muscle myosin isoforms, but not SMM. Similarly, A7r5 cells do not appear to express SMM (Kennedy et al. 2014). More recently, human induced pluripotent stem cells (iPSCs) have been successfully used to generate smooth muscle cells (reviewed in (Kwartler and Esparza Pinelo 2024). These cells have shown expression of SMM and have the potential to be studied both as a 2D monolayer and as 3D organoid models, holding promise for the future.

Studies on smooth muscle have been further complicated by the use of a wide range of tissue samples used to study intact smooth muscle, from chicken gizzard (Cande et al. 1983; Draeger et al. 1990; North et al. 1994; Small et al. 1986), rabbit portal-anterior mesenteric vein (Ashton et al. 1975), vas-deferens (Bond and Somlyo 1982), sheep airway and pulmonary arterial smooth muscle and rabbit carotid arterial smooth muscle (Liu et al. 2013), rat anococcygeal muscle (Godfraind-De Becker and Gillis 1988) and many others. It is not clear if or how variable the organisation of the contractile cytoskeleton across the smooth muscle types.

The focus of this review is to describe the properties and interaction of some of the key smooth muscle contractile proteins, to understand how they contribute to smooth muscle contraction, and their roles in disease.

## Introduction to myosin and actin and regulation of contraction in smooth muscle

**Fig. 1:**
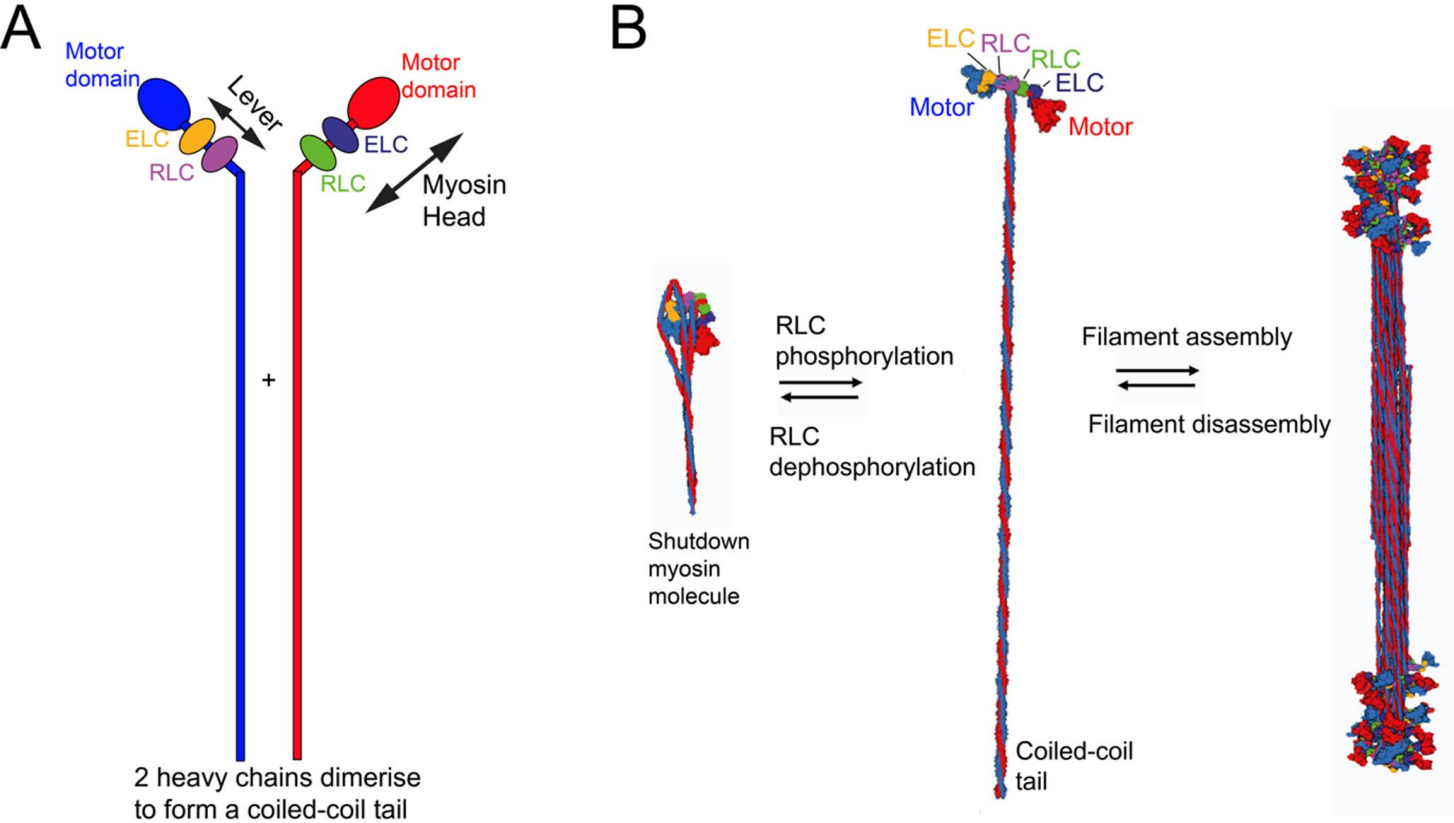
Basics of smooth muscle myosin. **A**: Schematic diagram of the two heavy chains (red and blue), and how they are divided up into motor, lever and tail domains. The light chains bind to the lever. The myosin head is comprised of motor, lever and light chains. B: SMM molecules, in which the RLCs are dephosphorylated, form a shutdown compact molecule, in which the two myosin heads interact with each other, and the coiled coil wraps around the heads. Phosphorylation of the RLCs in the shutdown myosin molecule disrupts the shutdown state, allowing the resulting extended molecule to assemble into filaments

Contraction in smooth muscle cells is driven by the interaction of smooth muscle myosin (SMM), organised into filaments, with filamentous smooth muscle actin (SMA), key components of the contractile cytoskeleton. SMM is a hexamer, comprised of two heavy chains, and four light chains: two regulatory light chains (RLC) and two essential light chains (ELC). (Fig. 1A). The heavy chains fold up to form the motor, which binds to actin and to nucleotide, followed by the lever, to which RLC and ELC bind. The heavy chains then dimerise by forming a coiled-coil tail, the distal third of which is important for filament formation. The contractile cytoskeleton is linked to the plasma membrane via the non-muscle actomyosin cytoskeleton, comprised of non-muscle myosin and actin isoforms, to transfer force to adjacent cells and/or the extracellular matrix through focal adhesions (or dense plaques: reviewed in (Zhang et al. 2023)).

The organisation of muscle actin and myosin into the contractile cytoskeleton is markedly different between striated (heart and skeletal) and smooth muscle. Striated muscle is so-called because of its stripy appearance. Actin and myosin are organised into thin and thick filaments, respectively, the lengths of which are regulated. The filaments are organised into muscle sarcomeres that have a consistent length, bounded by Z-discs at either end, Sarcomeres are organised in series along the length of muscle myofibrils, and connections between the Z-discs of adjacent myofibrils, as well as between the M-lines in the central region of the sarcomere, align the sarcomeres with each other across myofibrils across the muscle cell. This alignment across the fibre is responsible for the stripy appearance of the striated muscle cells. Force is transferred to the plasma membrane during contraction both longitudinally along the myofibrils to the end of the muscle cell, and laterally, through connections to the non-muscle myosin cytoskeleton in focal adhesion-like structures known as costameres that align with Z-discs.

In smooth muscle, assemblies of contractile protein filaments are much less well organised and are not connected and aligned as they are in striated muscle cells. This means the muscles look ‘smooth’ instead of striated. Structures somewhat equivalent to Z-discs, termed dense bodies are present, but not aligned with each other. The ratio of actin to myosin filaments in smooth muscle cells is more variable compared to that in striated muscle. It can vary from 8:1 (gizzard smooth muscle) to 15:1 in vascular smooth muscle, compared to 2:1 (by mass) in striated muscle (Gunst and Tang 2000).

In striated muscle, until recently, the standard explanation for its activation by an increase in cytosolic Ca^2+^, mainly released from Ca^2+^ stores in the sarcoplasmic reticulum, was by regulating available myosin binding sites on F‑actin by tropomyosin. The released Ca^2+^ binds to troponin in the thin filament, leading to a movement of the thin filament protein tropomyosin, which in turn exposes binding sites for myosin on actin, to allow contraction. However, there is now a greater emphasis on the role of regulating myosin activity as part of the regulation of contraction of striated muscle (recently reviewed in (Irving 2025). Many myosin heads in the thick filament in striated muscle adopt an interacting-heads motif (IHM), most recently seen in situ by cryo-electron tomography of isolated thick filaments and myofibrils (Dutta et al. 2023; Tamborrini et al. 2023). These heads likely have a lower ATPase activity helping to conserve energy in striated muscles (Stewart et al. 2010). Activation of muscle involves release of IHM heads in the thick filament (reviewed in (Irving 2025).

In smooth muscle, contraction is initiated by an increase in cytosolic Ca^2+^ as in striated muscle. However, much of the increase in Ca^2+^ is due to Ca^2+^ entry from the cell exterior through L-type calcium channels, with less from internal stores (sarcoplasmic reticulum), which are much less extensive in smooth than in striated muscle. In contrast to striated muscle, the main emphasis has been on myosin-based regulation as the main mechanism for regulating contractility in smooth muscle. The increase in Ca^2+^ activates the myosin light chain kinase (MLCK) by Ca^2+^-calmodulin, which then phosphorylates the regulatory light chain (RLC) of SMM. RLC phosphorylation directly activates SMM allowing it to interact with actin in the thin filaments (reviewed in (Ito et al. 2022) (Fig. 1). Relaxation of smooth muscle is driven by the reversal of RLC phosphorylation by myosin light chain phosphatase. The RhoA/ROCK signalling pathway can modulate this by inhibiting the phosphatase (reviewed in (Randar et al. 2025).

RLC phosphorylation could activate myosin already pre-assembled into filaments or activate isolated shutdown molecules that then assemble into new filaments (Fig. 1B), as dephosphorylated SMM can be found either pre-assembled into thick filaments (Somlyo et al. 1981) or as isolated shutdown molecules (Cross 1988) (Fig. 1B). In isolated shutdown molecules, the two myosin heads adopt an IHM as first described for smooth muscle myosin (Wendt et al. 2001). In these molecules, the coiled-coil tail wraps completely around the heads and the ATPase activity is very low (reviewed in (Cross 1988) (Jung et al. 2008a, b)). The compact nature of shutdown molecules allows them to be easily shuttled through the cytoplasm to generate new filaments when and where needed (reviewed in (Seow 2005)). As dephosphorylated SMM can be found in thick filaments it is also possible that the myosin heads could adopt an IHM in these filaments, although this has yet to be demonstrated in situ.

It is still not clear how much SMM is present in filaments, how much exists as shutdown molecules, and how this balance changes during contraction. In striated muscle, we know that most of the myosin is assembled into filaments. In smooth muscle, there is just one report claiming to show that there is a pool of shutdown myosin molecules, not organised into filaments (Milton et al. 2011). This was demonstrated using antibodies that could detect shutdown and filamentous myosin together with a peptide that inhibits the ability of myosin to remain as shutdown molecules. Indirect measurements such as muscle birefringence (Godfraind-De Becker and Gillis 1988) and measurements of levels of RLC phosphorylation (Chitano et al. 2017), have been used to report on a potential increase in filament formation on contraction. However, RLC phosphorylation assays would not differentiate between phosphorylation of SMM in isolated molecules and in filaments. Moreover, non-muscle myosin in smooth muscle cells is also activated by RLC phosphorylation in a very similar way to SMM (Casas-Mao et al. 2026; Cross et al. 1988).

In smooth muscle, the thin (actin-containing) filaments do not contain troponin, and so any activation of the thin filament during contraction is likely to be different from striated muscle. Both contractile and non-muscle actin filaments contain tropomyosin, and other proteins such as caldesmon and calponin can also be present. While skeletal muscle tropomyosin inhibits myosin ATPase activity, smooth muscle tropomyosin activates it (Marston and Smith 1985). The C-terminal domain of caldesmon (domain 4, discussed in more detail below) binds to tropomyosin in vitro and to F-actin and inhibits the actomyosin ATPase (reviewed in (Hodgkinson 2000)). Although a complex between caldesmon and tropomyosin on F-actin was not directly observed by electron microscopy, addition of a C-terminal caldesmon fragment (150 amino acids) changed the position of tropomyosin on actin (Hodgkinson et al. 1997). Phosphorylation of sites in the C-terminal domain of caldesmon (S759 and S789 in mammalian caldesmon) weaken its interaction for F-actin (reviewed in (Kordowska et al. 2006)). In addition, caldesmon has been reported to bind weakly to Ca^2+^-calmodulin, and in vitro, this binding can rapidly relieve caldesmon inhibition of the myosin ATPase (Marston 1986; Smith et al. 1987) (and see (Marston and Smith 1985) for a detailed discussion on this potential mechanism).

Calponin, like caldesmon, also inhibits the actomyosin ATPase, but works independently of tropomyosin (reviewed in (Hodgkinson 2000). Its binding to F-actin is Ca^2+^ independent, its ability to block contraction is relieved by phosphorylation, and it has been suggested to be more important in thin filament regulation than caldesmon (Winder and Walsh 1993). It is unclear if calponin and caldesmon can bind the same thin filaments in smooth muscle. Caldesmon has been reported only to be bound to actin filaments in the contractile cytoskeleton, while calponin may be restricted to the non-muscle cytoskeleton (Makuch et al. 1991), or have a more uniform distribution across smooth muscle cells (el-Mezgueldi 1996; Walsh et al. 1993). Overall, the detailed mechanism by which caldesmon and/or calponin might regulate the thin filament is not yet clear.

Finally, smooth muscle contraction may also involve an increase in actin polymerisation from a pool of monomeric (G-) actin. In one estimate, the amount of polymerised actin increased from about 70–80% of the total actin to about 90% (reviewed in (Gunst and Zhang 2008; Tang 2015). This increase in actin polymerisation likely includes both cytoskeletal actin (β-actin) and contractile filament actin (ɑ-actin) (Zhang et al. 2005). Changes to venous stiffness have been reported to arise from changes to the amounts of polymerised cortical actin (Saphirstein et al. 2015). The increase in actin polymerisation may be regulated by phosphorylation, with the Ste20-like kinase implicated recently in this process (Wang et al. 2020). This kinase regulates the phosphorylation of paxillin (a focal adhesion protein) by polo-kinase 1 to modulate N-WASP activation and actin polymerisation. Overall, more work is needed to determine what changes occur to the organisation of both the non-muscle cytoskeleton (non-muscle myosin and actins) and the contractile cytoskeleton (muscle actin and SMM) during contraction of smooth muscle.

## Smooth muscle contractile proteins

### Smooth muscle myosin, activation by phosphorylation and mutations

**Fig. 2:**
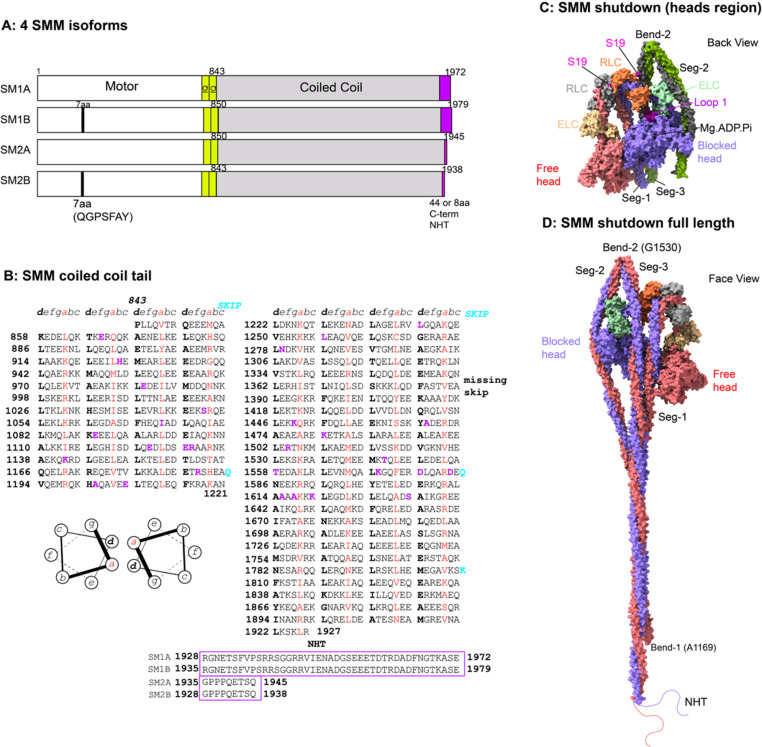
Smooth muscle myosin (SMM) heavy chain isoforms. **A**: Shows the four different isoforms. SM1B and SM2B have a 7 amino acid insert in the motor domain, and SM1A and SM1B have a longer non-helical tailpiece (NHT) as shown. **B**: shows the sequence for SM1A C-terminal coiled-coil tail, organised as heptad repeats (see inset diagram). The *a* (red font) and *d* (black, bold font) residues form the hydrophobic seam. The sequences of the C-terminal non-helical tail piece for each myosin are shown below. Residues shown in purple (bold) have been found to be mutated in disease (from the Human Genome Database), mainly resulting in thoracic aortic aneurysms. **C**: shows a surface view for the structure of SMM (PDB:6Z47) heads region. Both free and blocked head contain Mg. ADP. Pi in the nucleotide binding site. The position of loop 1 (the sequence of which varies between the isoforms) is indicated. The positions of Ser19 residues in the two RLCs in indicated. **D**: shows the pseudoatomic model for the full-length myosin (Scarff et al. 2020). This was not uploaded to the PDB, however the equivalent full-length model of NM2A is available on the PDB (PDB:9SZR). The models showing how the coiled coil wraps around the heads region, positions of the two bends, and three segments of coiled coil (Seg-1, -2 and − 3), and the position of the non-helical tail piece

The smooth muscle myosin (SMM) heavy chain is encoded by a single gene (*MYH11*). Its amino acid sequence is highly similar to that of the three non-muscle (NM) class 2 myosin isoforms encoded by *MYH10*,* MYH11* and *MYH14*. NM2A (encoded by *MYH9*) and NM2B (encoded by *MYH10*) heavy chains are also expressed in smooth muscle but would not be expected to co-polymerise with SMM. SMM forms long (~ 2.2 to 2.4 μm in length, ~ 15 nm wide) side polar filaments (Ashton et al. 1975; Somlyo 2015), whereas non-muscle myosin forms short (~ 0.3 μm) bipolar filaments (Billington et al. 2013). The expression of *MYH11* is restricted to smooth muscle (reviewed in (Babu et al. 2000)).

The N-terminal 842 residues of the SMM heavy chain form the myosin head together with the essential (ELC) and regulatory (RLC) light chains. This myosin head is comprised of an N-terminal SH3-like fold, the motor domain (which binds to actin and contains the nucleotide binding pocket) and the lever, which contains the converter domain followed by two ‘IQ’ (isoleucine, glutamine) repeats, each of which binds a light chain. The essential light chain (ELC) binds to the first IQ motif, following the motor domain. The regulatory light chain binds to the second. The remaining residues of the heavy chain, following the invariant proline (P843) residue dimerise the myosin via a coiled coil, apart from a small number of residues at the C-terminus, which are disordered in structure (non-helical tail piece).

There are four known variants of human SMM heavy chain (Fig. 2A). Two of these, SM1B and SM2B, have an additional 7 residues (QGPSFAY) inserted in the motor domain, in a region called loop 1, which lies just outside the nucleotide binding pocket. The sequence and length of this loop modulate the rate of ADP release; a longer loop increases the rate of ADP release (Sweeney et al. 1998). The two SM-B isoforms are mainly expressed in phasic smooth muscle that more rapidly contracts, which is found in small muscular arteries, bladder, intestine and stomach, where the ratio between these two isoforms can vary. The two SM-A isoforms are mainly expressed in tonic smooth muscle that generates slower, more sustained contractions, which is found in large arteries. Two SMM isoforms, SM1A and SM1B have a longer non-helical tail piece (NHT) than the other two (SM2A and SM2B). This region is important for filament assembly and may prevent heterodimerisation of SM1 and SM2 isoforms (Fig. 2B) (Rovner et al. 2002).

SMM and all three NM isoforms form a shutdown state as isolated molecules in which the myosin tail wraps around the head to form a compact structure (Fig. 2C, D) (Jung et al. 2008a, b) and the ATPase is very low (Cross et al. 1988). In contrast, skeletal and cardiac myosin only forms a partial shutdown state as isolated molecules, in which the coiled-coil tail does not wrap around the head completely (Jung et al. 2008a, b). Three high resolution Cryo-EM structures of shutdown smooth muscle myosin were reported recently (Heissler et al. 2021; Scarff et al. 2020; Yang et al. 2020) together with a new Cryo-EM structure for NM2A (Casas-Mao et al. 2026) revealing the details of interactions within these molecules that stabilise the shutdown state.

Release of SMM and NM2A from their shutdown state depends predominantly on phosphorylation of serine 19 in the RLC, by myosin light chain kinase. The CryoEM structure of shutdown isolated SMM molecules shows that the N‑terminal phosphorylation domain of the free head RLC (grey, Fig. 2C) lies between the two RLCs (a structure we named the mortar, as it appears to cement the two RLCs together) and that Ser19, which marks the start of this domain, is exposed and available for phosphorylation (Fig. 2C). The N-terminal phosphorylation domain of the blocked head RLC (orange, Fig. 2C) reaches towards Seg-3 of the coiled coil (a structure we named the latch) and Ser19 is more buried. We argued that phosphorylation would disrupt the mortar first, loosening the connection between the two RLCs, which lie just above the head-tail junction, to begin destabilising the shutdown molecule (Scarff et al. 2020). This then makes Ser19 available for phosphorylation in the blocked head RLC, to further destabilise the molecule. Our recent high-resolution structure for shutdown NM2A supports this idea (Casas-Mao et al. 2026). The molecule then extends and can assemble into filaments in which the myosin is active. The equivalent structural data for filamentous SMM is lacking. The residue Thr18 can also be phosphorylated. A Ca^2 +^ independent kinase, integrin-linked kinase, has been reported to phosphorylate this residue in smooth muscle (Sutherland and Walsh 2012), and drug treatment of smooth muscle elicits dephosphorylation, resulting in a sustained contraction and marked slowing of relaxation (Sutherland and Walsh 2012). Presumably, this is because both residues must become dephosphorylated to switch the myosin off.

**Fig. 3:**
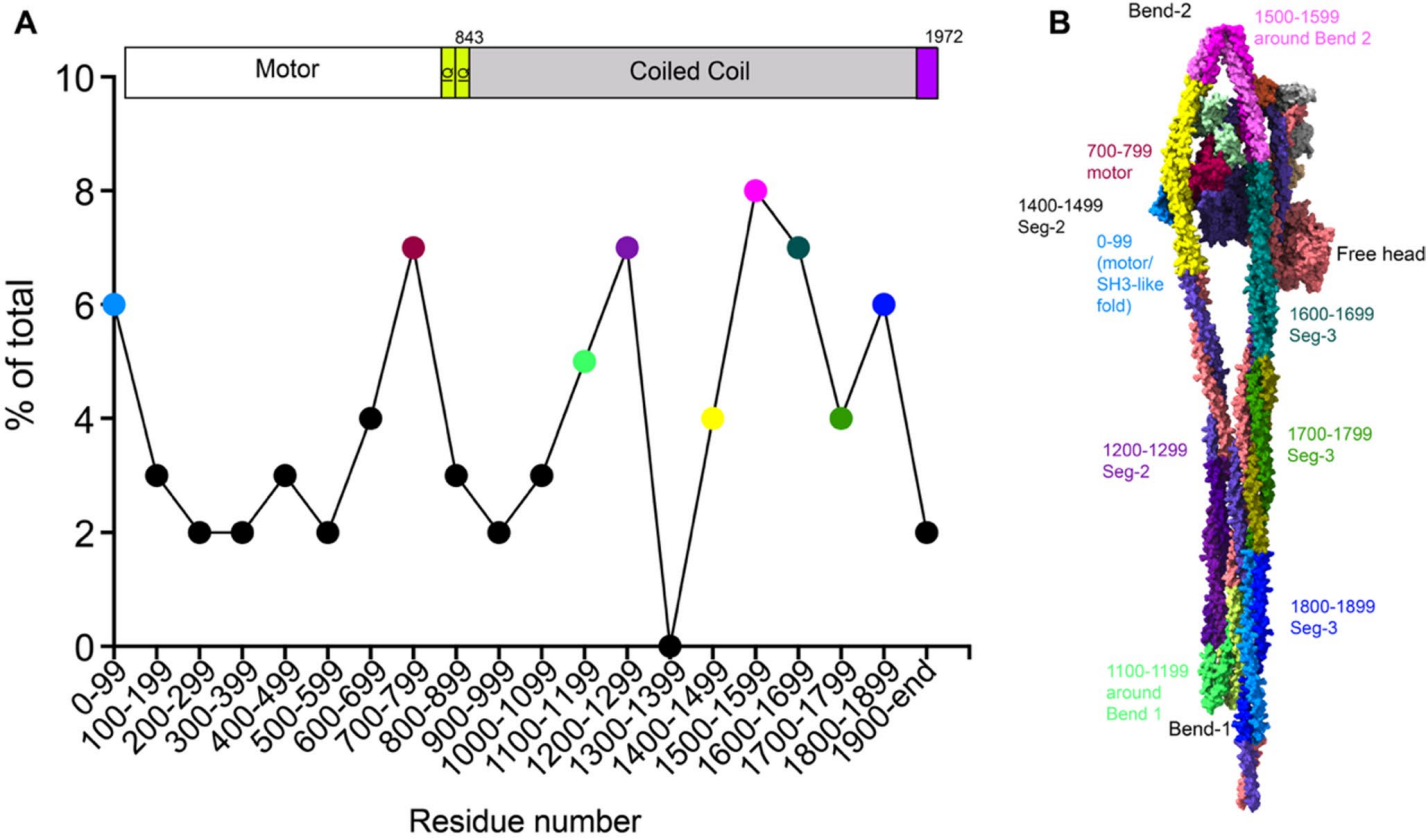
Mutations in SMM. **A**: shows the numbers of mutations for every 100 residues along the sequence, based on those reported in the HGMD database (accessed Nov. 2024). Over 70% of mutations lie in the coiled coil. The regions of coiled coil shown in **A** are identified in the full-length model in **B**, for Seg-2 (from Bend-1 to Bend-2) and Seg-3 (from Bend-2 to the end of the coiled coil) using the same colour scheme, with residue numbers as indicated. (List of missense mutations can be found in Table S1. Terminating mutations are excluded). Pseudoatomic model in B, not available on the PDB (see legend to Fig. 2)

About 80 missense mutations have been reported for *MYH11* in the HGMD (human genome mutation database, accessed Nov 2024). This database collates published mutations that occur in a range of genes. These mutations are predominantly associated with thoracic aortic aneurysm and dissection. Analysis of these mutations (Fig. 3A) shows hotspots in specific regions of the heavy chain. These include mutations in the N-terminal region including the SH3-like fold (between residue 1 and 99), and in the motor itself (between residues 700–799). These two regions directly interact with Seg-2 of the coiled coil (residues 1400–1499, shown in yellow) in the shutdown molecule (Scarff et al. 2020). Some of these mutations affect residues that directly interact with the coiled coil. One example is K78, which forms an ionic interaction with E1434 in Seg-2. The mutation K78E, associated with thoracic aortic aneurysms (Harakalova et al. 2013), reverses the charge of this residue and would disrupt this stabilising interaction. Mutations in the equivalent residues (K74E and E1421K) in NM2A, cause MYH9-related disease (Kanematsu et al. 2016) and (Zaninetti et al. 2019). Overall, mutations in this region are likely to destabilise the shutdown state, as we showed experimentally for NM2A (Casas-Mao et al. 2024). A high-resolution structure of SMM would greatly help in understanding the impacts of specific mutations. Although a higher resolution structure (~ 3.2 Å: PDB 7MF3) has been reported for the heads region (Heissler et al. 2021), unfortunately, the coiled coil structure unexpectedly goes out of register in that structure, likely because the structure was not built correctly into the EM density map (Casas-Mao et al. 2026). This makes it difficult to use to interpret the effects of mutations in the coiled coil using that structure.

Over 70% of the mutations in the SMM (heavy chain) are found within the coiled-coil tail. Eight mutations lie between residues 1500–1599, which lie either side of Bend 2 (Fig. 3B), and could destabilise the shutdown state, by affecting bend formation. 19 mutations are found in the coiled coil from residue 1600 onwards (the distal region of Seg-3), eight between residues 1100 and 1300 (the proximal region of Seg-2) and 3 between residues 1000 and 1100 (the distal region of Seg-1). Thus, over 40% of mutations are in regions of the coiled coil that interact with each other below the interacting heads region in the same molecule in the shutdown state and likely interact with adjacent molecules within filaments. In the shutdown state, we found that ionic interactions between Seg-1 and − 3 were staggered by about 107 nm along the coiled coil (Scarff et al. 2020), similar to that predicted to occur in thick filaments (Straussman et al. 2005). This suggests that mutations in this region of the coiled coil could both disrupt shutdown molecules and interfere with filament formation. Smooth muscle contraction would likely be reduced through dysregulation of the equilibrium between shutdown myosin and filaments, and through effects on myosin filament organisation.

### Smooth muscle actin and mutations

The human genome encodes 6 actin isoforms (Parker et al. 2020). *ACTA2* encodes one of the three ɑ-actin isoforms (ACTA: aortic smooth muscle ɑ-actin isoform) and is expressed in smooth muscle. The other two ɑ-actin genes (*ACTC1* and *ACTA1*) are specific for cardiac and skeletal muscle respectively. *ACTG2* encodes one of the two ɣ-actin isoforms (ACTH, ɣ -enteric smooth muscle), and its expression is also specific to smooth muscle. ACTA is predominantly expressed in vascular smooth muscle and ACTH in gastrointestinal smooth muscle (reviewed in (Lehman and Morgan 2012). The two non-muscle isoforms; β- and ɣ-actin, encoded by *ACTB* and *ACTG1* respectively are both widely expressed, including in smooth muscle cells, where they assemble into the non-muscle cytoskeleton. The two muscle isoforms, ACTA and ACTH assemble into the equivalent of muscle thin filaments (reviewed in (Lehman and Morgan 2012) in smooth muscle cells.

**Fig. 4:**
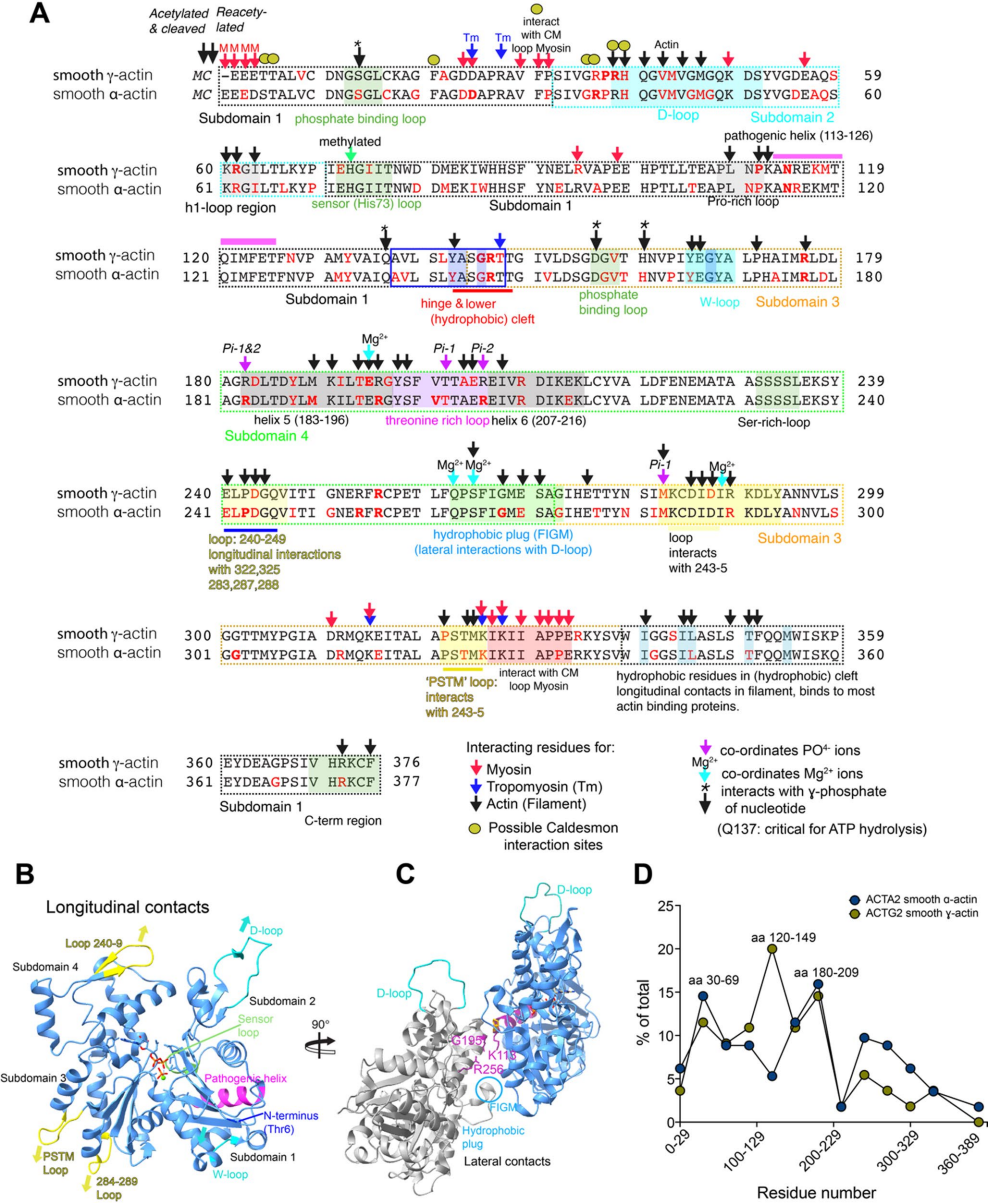
**A**: Sequence alignment between the two smooth muscle actin isoforms: smooth muscle ɣ-actin (ACTG2) and smooth muscle -actin (ACTA2). The 4 subdomains in actin are indicated by dashed boxes. Areas of interaction with myosin, tropomyosin are indicated (see Key). Residues reported in HGMD (Human Genome Mutation Database) reported to be missense mutations are indicated in red, and in bold red if 2 or more missense occur in the same residue. Terminating mutations are excluded. **B**: The structure of a single actin subunit from an actin filament structure (PDB: 8RU0; (Oosterheert et al. 2024) showing the 4 subdomains, specific loops involved in longitudinal contacts along a single strand of actin monomers in the filaments, and the pathogenic helix and sensor loop in relation to Mg^2+^ ATP in the central region of the molecule. **C**: shows two subunits from the two separate strands in the filament (PDB: 8RU0) to show the lateral interactions between the two strands. (List of missense mutations can be found in Table S2 & 3). D. A plot to show the frequency of mutations in ACTA2 and ACTG2: The number of missense mutations in groups of 30 residues (e.g. 0‑29, 30–59 and so on) were counted and then expressed as a percentage of the total number of mutations (113 for ACTA2, 55 for ACTG2). (List of missense mutations can be found in Tables S2 & 3. Terminating mutations are excluded)

The numbers of mutations reported for the smooth muscle thin-filament actin isoforms, ACTA and ACTH, have risen over the past 5 years since we last reviewed mutations in all 6 actin genes (Parker et al. 2020) (Fig. 4). The number of missense mutations has risen from below 90 to 110 in ACTA (HGMD database and associated publications; Table S1), most of which are associated with thoracic aortic aneurysms. The number of missense mutations in ACTH have risen from 20 to 50, and these mostly cause disease in the intestinal smooth muscle (e.g. megacystis-microcolon-intestinal hypoperistalsis syndrome; Table S1).

Many of these actin mutations lie in critical sites important for polymerisation (Fig. 4A and B) as we found previously (Parker et al. 2020). Although the numbers of mutations have risen, they still show a somewhat similar pattern for hotspots. One set of hotspots for mutations affect key regions of actin important for monomer: monomer (subunit: subunit) interaction along one of the two helical strands. These include missense mutations in the ‘D’ loop, ‘PSTM’ loop, loop 284–289 and the W-loop (Fig. 4A, B & D). Oher missense mutations in residues in the hydrophobic plug (FIGM) and either close to, or in residues K113, G195 and R256 that make lateral interactions between monomers across the two helical strands are also likely to affect polymerisation and depolymerisation (Fig. 4A, C & D).

Smooth muscle cells modulate both actin and myosin filament levels in response to external signals, as discussed earlier. Mutations that affect actin filament stability will impact filament formation and organisation and affect the ability of actin to form additional filament, when required, explaining why mutations in areas critical for polymerisation are common. It is also interesting to see that M46 is mutated in ACTA, as oxidation of this residue by MICAL (molecule interacting with CasL), results in rapid actin depolymerisation, important for remodelling the actin cytoskeleton (Rajan et al. 2023). We found that this residue is mutated in all 6 actin isoforms (Parker et al. 2020). Moreover, mutations in residues that co-ordinate and respond to the presence of nucleotide in the cleft between subdomains 1 and 2, and subdomains 3 and 4, are also hotspots for mutations (Fig. 4A). The nucleotide state of actin is again important in polymerisation, with new actin monomers adding to the barbed ends of filaments as ATP-actin and leaving from the pointed end as ADP-actin. Thus, most of the missense mutations in smooth muscle actin are most likely to affect polymerisation dynamics.

Myosin and actin filaments need to be organised in a way that enables muscle contraction. In striated muscle, the length of thick (myosin-containing) filaments is regulated by the protein, titin, which runs from the Z-disc, through into the thick filament, terminating near/at the M-line. Myosin-binding protein C (MyBPC) is also present in the thick filaments, in the central C-zone, and helps to regulate myosin activity. The organisation of these proteins (myosin, MYBPC and titin) in the C-zone of thick filaments has recently been demonstrated by cryo-electron tomography (Chen et al. 2024; Dutta et al. 2023; Tamborrini et al. 2023). Both skeletal and cardiac muscle thin (actin-containing) filaments additionally contain tropomyosin and troponin, regulatory proteins that control the availability of actin binding sites for myosin. In skeletal muscle, nebulin is also present in thin filaments, and helps to stabilise and regulate its length, while a shorter related protein, nebulette, is found in cardiac muscle. A cryo-electron tomography dataset has revealed the organisation of many of these proteins in the thin filament, and demonstrated how nebulin might act as a molecular ruler in regulating thin filament length in skeletal muscle (Wang et al. 2022). Its absence in cardiac muscle is associated with a more variable thin filament length. 

### Smooth muscle titin

In smooth muscle, many of the proteins found in thick and thin filaments in striated muscle are absent. Thick filaments do not contain MyBPC, and thin filaments do not contain troponin or nebulin. However, two alternatively spliced forms of titin are present in smooth muscle, demonstrated by both RT-PCR and western blotting (Labeit et al. 2006). One of these isoforms is about 1MDa in size and expressed predominantly in adult aorta. The other is larger, similar in size to that expressed in slow muscle fibres (~ 2-3.7MDa) (Labeit et al. 2006). Titin expression levels are about 10x lower in smooth muscle than in striated muscle cells, perhaps not unexpected given the 5x lower levels of smooth muscle myosin. Smooth muscle titin could link myosin in thick filaments to dense bodies (equivalent to Z-discs in striated muscle) and potentially play some role in organising smooth muscle myosin thick filaments. Splicing to generate different titin isoforms from the single titin (TTN) gene, is partly dependent on the ribosomal protein RMB20, which promotes exclusion of coding exons for the I-band (actin only region) in both striated and smooth muscle (Zhu et al. 2025). A recent study showed that the deletion of RBM20 in rat smooth vascular smooth muscle, promoted expression of the longer titin isoform in these cells, and was linked to a reduced cellular stiffness (Zhu et al. 2025).

The organisation of titin in smooth muscle cells, and its relationship to myosin filaments has yet to be studied in detail. Is it able to regulate thick filament length in smooth muscle, as it does in striated muscle cells? How would this work? Myosin organisation in thick filaments in striated muscle is highly regulated with exactly 294 molecules per filament, and a length of 1.6 μm. The myosin molecules are organised in a bipolar fashion, with antiparallel packing of the coiled-coil tail molecules in the central M-line, and parallel packing throughout the rest of the filament. The C-terminal region of titin is found at the M-line, where it interacts with additional M-line proteins such as myomesin (Lange et al. 2020). However, in smooth muscle, myosin likely assembles into side‑polar filaments (Craig and Megerman 1977; Xu et al. 1996). In these filaments, the myosin heads, with 4 molecules per 14.5 nm, project from opposite sides of the filament (two on each side), there is no central bare-zone and the filaments are ~ 2.2 μm long (thus ~ 600 molecules per filament) (Somlyo 2015). As yet, there is no high-resolution imaging data for smooth muscle thick or thin filaments in situ that could reveal the details of their organisation.

### Smooth muscle tropomyosin

**Fig. 5:**
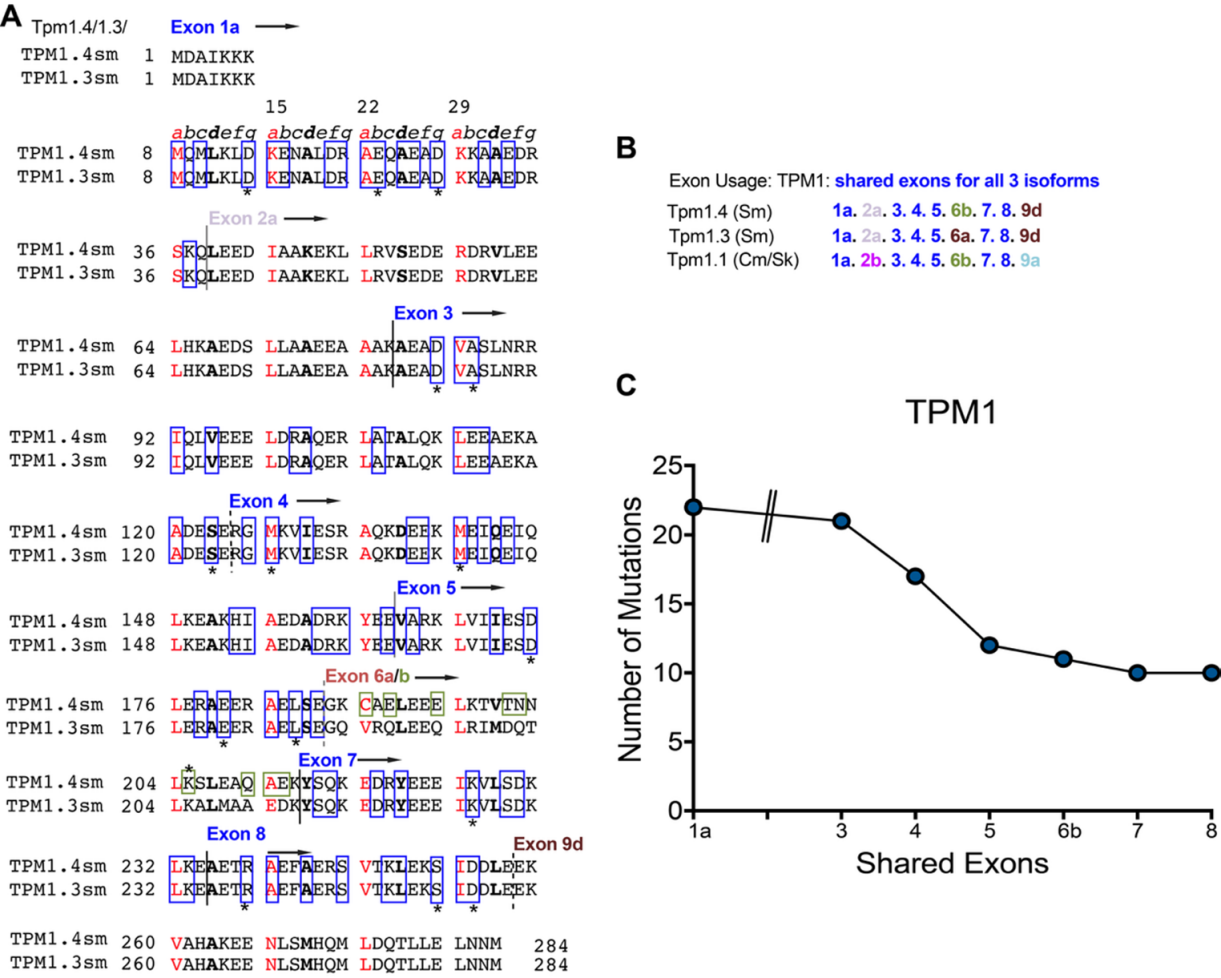
Alignment of the two of the three smooth muscle isoforms of tropomyosin (TPM1.4 and TPM1.3). The positions of *a* and *d* residues in the heptad repeat are indicated. The positions of each exon coding region are indicated, with dotted lines for splicing that is not in frame, and solid for in frame. Exons shared with Tpm1.1, which codes for an isoform of tropomyosin found in striated (cardiac and skeletal) muscle are indicated in blue font, with exon 6b shared between TPM1.4 and 1.1 only, shown in green. Positions of missense mutations in the protein encoded by shared exons in the TPM1 gene (obtained from HGMD) are indicated by the blue coloured boxes, except for Exon 6b, where they are shown in green. Residues with more than 1 mutation are indicated by an asterisk. (The third SMC isoform, TPM2.1, is not shown**). B**. Exon usage by Tpm1.3 and 1.3 (smooth muscle) and Tpm1.1 (striated). **C**: Plot to show numbers of mutations per exon, for the shared exons between TPM1.1 and TPM1.4 and/or TPM1.3. (Mutations listed in Table S4)

Tropomyosin is present in smooth muscle thin filaments (reviewed in (Marston and Smith 1985), but troponin and nebulin are absent. Tropomyosin is a dimer formed by two ɑ-helices that wrap around each other. Tropomyosin isoforms expressed specifically in smooth muscle include Tpm1.3 (ɑ-smooth tropomyosin, or Tm sma-1) and TPM1.4 (ɑ-smooth tropomyosin, Tm smɑ, or Tm6) both of which are alternatively spliced from the *TPM1* gene, and TPM2.1 (Tm smβ, βTM TM1) derived from the *TPM2* gene (described in (Geeves et al. 2015)). All three isoforms are 284 amino acids long and show approximately 70% similarity at the amino acid level. TPM1.3 and TPM1.4 share exon usage with striated muscle tropomyosin TPM1.1 but contain sequence from the unique 2a exon only found in smooth muscle (Fig. 5). There are over 100 missense mutations in the *TPM1* gene, most of which are associated with either hypertrophic or dilated cardiomyopathy. Missense mutations in the *TPM2* gene (about 30) are mostly associated with skeletal muscle diseases (predominantly nemaline myopathy). It is possible that some of the mutations in *TPM1*, present in shared exons, will affect smooth muscle TPM isoforms (Fig. 5A, B) and could affect the contractile cytoskeleton of smooth as well as cardiac muscle. The effects of those mutations could also contribute to the heart phenotype, as heart is highly vascularised. One of the key roles of tropomyosin in smooth muscle is to help stabilise the actin filaments, and thus mutations have the potential to decrease thin filament stability in the smooth muscle cells, affecting their contractility.

### Smooth muscle caldesmon

**Fig. 6:**
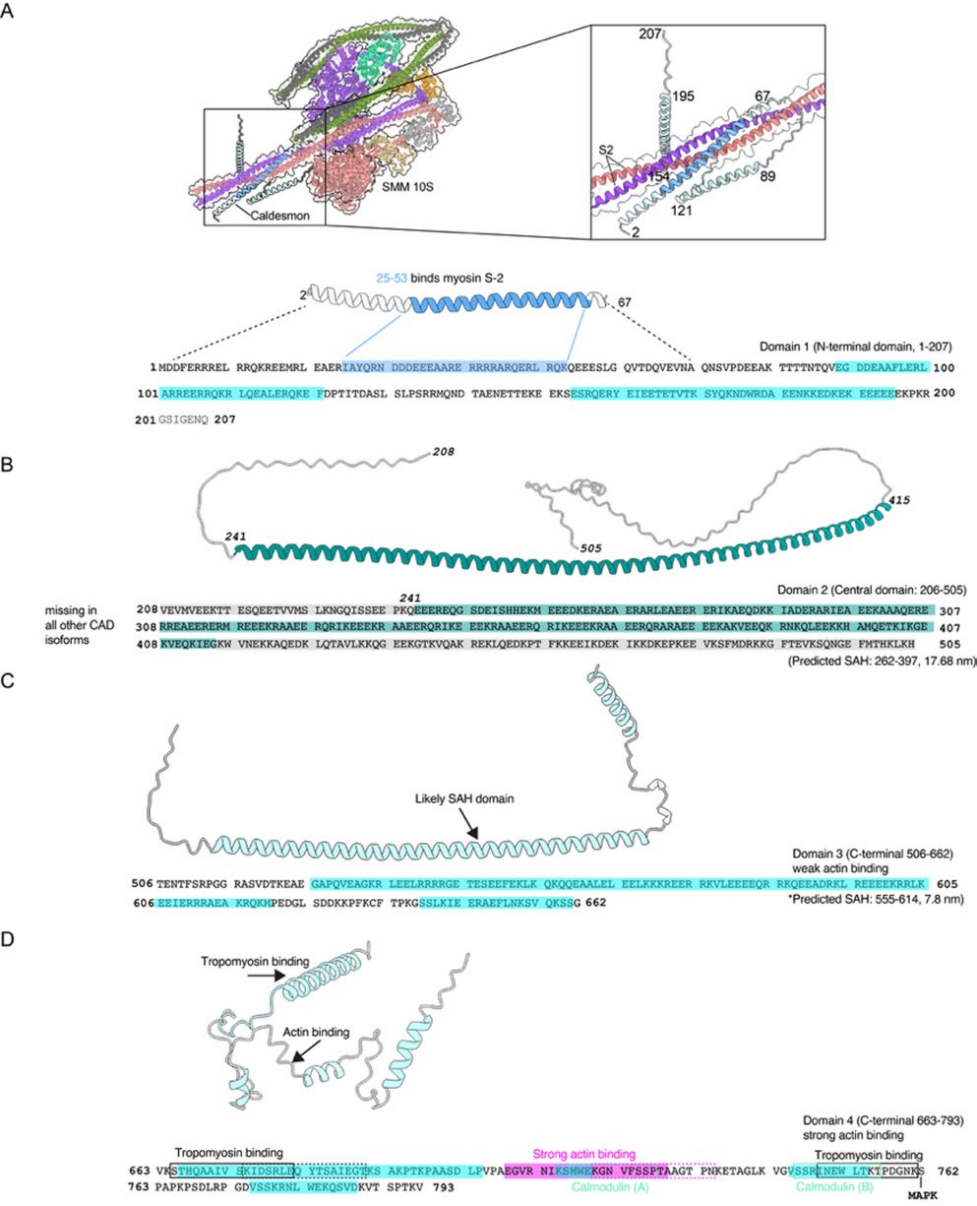
The sequence and ɑ-helical regions of H-caldesmon. **A**. Domain 1 contains a region of ɑ-helix that likely binds to the S-2 (the first part of the coiled coil: Subfragment-2), predicted to be in the region just below the interacting heads in shutdown SMM, as shown. Alphafold3 predicted structure for this region of caldesmon, together with structure of SMM (PDB:6Z47 (Scarff et al. 2020) (Fig. S2A, B). **B**: Sequence and the Alphafold3 predicted structure for domain 2 (Fig. S2C) predicts a long single ɑ-helix, only found in this long form of caldesmon. The presence of this long single ɑ-helix has been demonstrated experimentally. **C**: Sequence and Alphafold3 predicted structure (Fig. S2D) of domain 3, which is predicted to bind weakly to actin. Waggawagga (Simm et al. 2015) also predicts most of this region to be a second SAH) domain (Fig. S1). **D**: Sequence and Alphafold predicted structure of domain 4, which contains tropomyosin, calmodulin and strong actin binding sites, as shown (This prediction is the least likely to be correct, as predicted by Alphafold: Fig. S2E)

The protein H-caldesmon, found specifically in smooth muscle, is encoded by the *CALD1* gene, which also encodes several other shorter isoforms found in non-muscle cells. There is likely to be only one caldesmon molecule for every fourteen or so actin monomers in the filament, and it has been suggested to modulate smooth muscle contraction (as discussed above). Caldesmon isoforms show some variation in sequence in the first few residues at the N-terminus but the key difference between H-caldesmon and shorter isoforms is that H-caldesmon uniquely contains a long, repetitive sequence in the central region (domain 2) of the protein that forms a stable single ɑ‑helix (SAH) approximately 34 nm long (Peckham and Knight 2009; Wang et al. 1991) (Fig. 6B:, Fig. S2C). The full length molecule has been estimated to be 75 nm long (Hodgkinson 2000). The SAH domain can also be predicted using Waggawagga (Fig. S1) (Simm et al. 2015).

The first 67 residues of the N-terminal domain (domain 1) are likely helical (as predicted by Alphafold3; Fig. S2A), and the central region of this sequence likely binds the first part of the coiled coil (S-2, or the first part of Seg-1) (Fig. 6A; Fig. S2B). Domain 3 of caldesmon (Fig. 6C), which is shared by both smooth muscle and non-muscle forms of caldesmon, is also likely to form a short SAH domain, from inspecting its sequence, Alphafold modelling (Fig. S2D) and from using Waggawagga, which accurately predicts the presence of SAH domains (Fig. S1). This domain could also contribute to the observed bridging between thick (myosin) and thin (actin) filaments in both smooth and non-muscle cells, as does domain 2 (Kokate et al. 2022; Marston et al. 1992). Consistent with this idea, the shorter form of caldesmon acts as a dynamic crosslinker between non-muscle myosin filaments and actin bundles in the stress fibre network of non-muscle cells (Kokate et al. 2022). Caldesmon has also been shown to bundle actin filaments both in smooth muscle and non-muscle cells (Kokate et al. 2022; Moody et al. 1985). Domain 4 contains the actin and tropomyosin binding sites (Fig. 6D) (Marston et al. 1994). Thus, caldesmon can span across the thick and thin filaments, perhaps analogous to the ability of MyBPC to do so in striated muscle.

### Smooth muscle calponin, transgelin and smoothelin

Calponin, transgelin (originally known as SM22) and smoothelin are three further highly expressed proteins found in smooth muscle cells. There are three isoforms of calponin, an actin binding protein that can modulate the dynamics of the actin cytoskeleton, of which calponin 1 is specific to smooth muscle cells (Liu and Jin 2016). It contains a calponin homology (CH) domain that binds tropomyosin, followed by two actin binding sites that can bind actin and gelsolin. This binding can inhibit the nucleating ability of gelsolin (Ferjani et al. 2006). The C‑terminal region (which includes a second actin binding site) has also been reported to bind microtubules (Liu and Jin 2016). Transgelin is homologous to calponin, is highly abundant in smooth muscle cells and plays a similar role in regulating the stability of the actin cytoskeleton as does calponin (Hsieh and Jin 2023). Smoothelin, of which there are two isoforms, A and B, as well as the related smoothelin-like proteins are again linked to modulating or regulating smooth muscle contractility (reviewed in (Murali and MacDonald 2018).

### Smooth muscle potential protein-protein interactions: in the contractile cytoskeleton

**Fig. 7:**
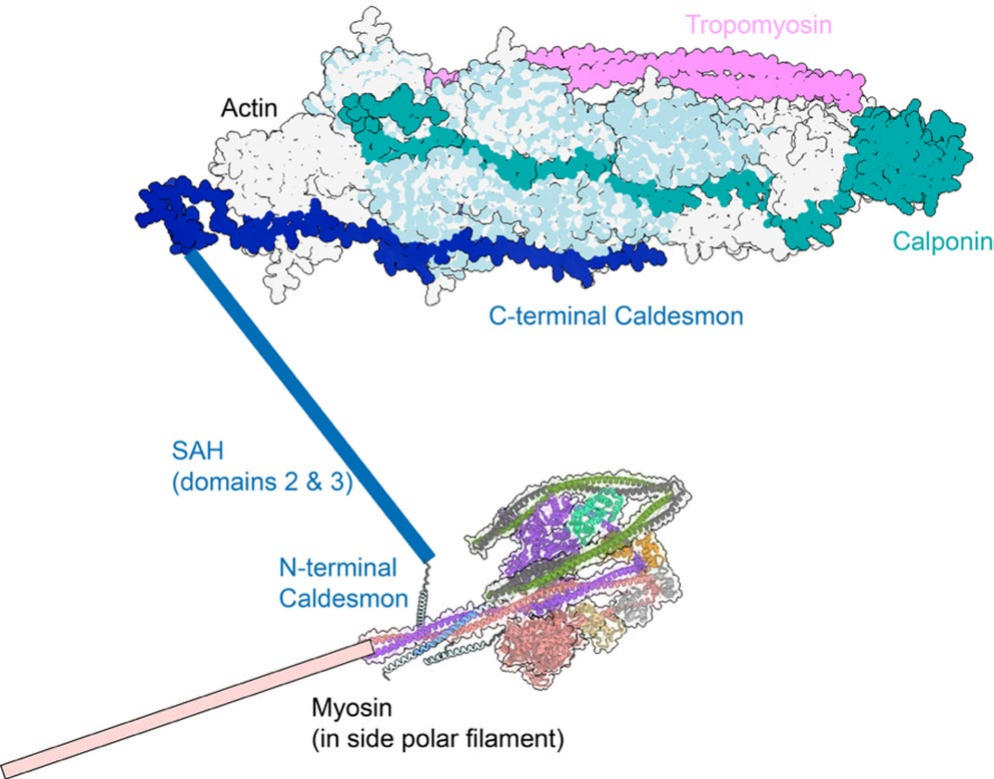
Diagram to show where caldesmon and calponin may lie in the thin filament, and how caldesmon bridges across to the thick filament to interact with myosin. The C-terminal region, calponin and F-actin were modelled using Alphafold (Fig. S3). As Alphafold modelled tropomyosin poorly (as assessed by a visual inspection), tropomyosin in the Alphafold model was replaced by tropomyosin on F-actin from a cryoEM structure reported previously (PDB: 5NOG) (Risi et al. 2017) using ChimeraX (https://www.rbvi.ucsf.edu/chimerax). The N-terminal of caldesmon is associated with the S-2 region of myosin (Fig. 6A). The pink rectangle indicates the rod domain of smooth muscle myosin (coiled coil) that would lie within the myosin filament. The central SAH domain of calponin is shown as a blue rectangle. The C-terminal caldesmon is shown in its predicted region on the thin filament (as predicted by Alphafold modelling). Calponin is predicted (by Alphafold3) to run in the groove between the two actin strands in the filament. (Diagram not to scale)

The potential organisation of most of these proteins can be predicted through Alphafold modelling (Fig. 7). A key point here is that interactions between proteins demonstrated in vitro, typically only investigate the two proteins of interest. Possible interactions observed in vitro, might be blocked or absent in the thin filament complex. Likewise, Alphafold3 predictions for such complexes may not be accurate (Fig. S3), and any predictions need to be tested experimentally. Alphafold modelling failed to provide a clear picture of how tropomyosin might assemble into the thin filament complex, for example. Here, a structure for tropomyosin bound to actin (Risi et al. 2017) was overlaid on the Alphafold model for the protein complex (Fig. S3), and used to replace tropomyosin in the model structure.

The Alphafold model predicted that calponin lies in a similar groove on F-actin as experimentally observed for nebulin bound to skeletal F-actin ((Wang et al. 2022) (Fig. S3). Interestingly, it failed to show an interaction between calponin and tropomyosin. Moreover, the N-terminal domain of calponin was either seen to bind to the end of the filament (Fig. 7), or between actin monomers in the filament (Fig. S3), which might explain its ability to interfere with the binding of gelsolin. The folding back of C-terminal region of calponin observed (Fig. 7) is likely because the model shown here only contains 8 actin monomers (due to restrictions in setting up the model in Alphafold). Individual models for F-actin and caldesmon or F-actin and calponin showed similar arrangements as in the combined model (Fig. S3, B, C). A further model for F-actin, caldesmon and calmodulin showed the binding of calmodulin to caldesmon, which was the same as the strong binding region shown experimentally (Fig. 7, Fig. S3D).

Despite potential issues with the Alphafold modelling, it does raise interesting questions. Does calponin really lie in a similar position to nebulin on F-actin and thus play a role in stabilising actin filaments? Does the C-terminal of caldesmon lie along F-actin and if so, how does it interact with tropomyosin in situ? There is clearly much we have yet to understand about the structure and organisation of the contractile thin filaments in smooth muscle.

## Dense bodies

In striated muscle, longitudinal forces are relayed by the Z-discs, structures that anchor the thick and thin filaments in the muscle sarcomeres, arranged longitudinally along the myofibrils. The barbed ends (fast growing ends) of the thin filaments are anchored in the Z-discs. Z-discs are complex structures that contains over 50 different proteins, both structural and signalling. Similarly, myofibrils are anchored to the membrane at the level of Z-discs by costameres providing lateral force transmission (Luther 2009).

In smooth muscle, the thick (~ 12–15 min diameter) and contractile thin filaments (~ 7 nm in diameter) are anchored by cytoplasmic dense bodies, which also incorporate intermediate filaments (~ 10 nm in diameter). Dense bodies are found throughout the cytoplasm, and, unlike Z-discs in striated muscle, are not aligned to each other. In vascular smooth muscle, the intermediate filaments are mostly made up of vimentin, and in airway muscle, desmin (Gunst and Tang 2000). As found for Z-discs, the barbed ends of thin filaments are located within the cytoplasmic dense bodies (Bond and Somlyo 1982; Gunst and Tang 2000). Thus, the cytoplasmic dense bodies can be considered somewhat equivalent to Z-discs in striated muscle. Cytoplasmic actin filaments are anchored by dense bodies (plaques) found at the plasma membrane in smooth muscle cells, which are like focal adhesions in non-muscle cells.

The constituents of the cytoplasmic dense bodies in smooth muscle cells are poorly characterised. It seems likely that capping protein caps the thin filaments within the dense bodies, to prevent further growth of the filaments, as in striated muscle Z-discs. Capping protein is a heterodimeric protein comprised of ɑ and β subunits, that binds to the barbed end of actin filaments, preventing growth and loss of actin monomers from this end of the filament. The presence of CapZ, the muscle specific from of capping protein found in Z-discs (Casella et al. 1987) has been reported for saphenous vein smooth muscle cells, and its expression linked to hemodynamic stress (McGregor et al. 2004). The muscle isoform of the cross-linking protein ɑ-actinin (ACTN2) has also been reported to be associated with dense bodies (Lohanadan et al. 2023), as it is in Z-discs in striated muscle, and will likely crosslink the actin filaments in these structures. Smooth muscle titin also interacts with the rod domain of ACTN2 in the dense body through a specific motif (Zq) in its N-terminal region (Chi et al. 2008) as well as a further interaction with the C-terminal domain of ACTN2. Very few other proteins have been described. One is synaptopodin 2 (SYNPO2, myopodin, fesselin), which can accelerate actin filament polymerisation (Chalovich and Schroeter 2010) and interacts with ACTN2 and synemin, a member of the intermediate filament family, potentially linking dense bodies to intermediate filaments (Lohanadan et al. 2023). Specific splice isoforms of SYNPO2 are expressed in heart, skeletal and smooth muscle (Lohanadan et al. 2023). Another Z-disc related protein, filamin (FLNa), is also found in dense bodies in smooth muscle, although is excluded from the core (Small et al. 1986). It is possible that by having fewer proteins in dense bodies compared to Z-discs, allows them to be more dynamic (form and disassemble more rapidly) and results in their less regular shape.

## Concluding remarks

Smooth muscle cells are somewhat understudied compared to skeletal and cardiac cells, likely because a good cell line that recapitulates the full smooth muscle phenotype is lacking, due to the plastic nature of these cells. Primary cells must be used and are not easy to obtain. Similarly, the contractile protein organisation in smooth muscle tissue is highly variable and researchers have used a broad range of tissues in their studies, that can make it challenging to compare experiments. Surprisingly, much of the literature for the smooth muscle contractile and non-muscle cytoskeleton is relatively old. There are still many outstanding questions, from determining the precise components and organisation of proteins in dense bodies, to determining how much of the actin and myosin molecules exist in filaments, or as individual monomers, how this varies between smooth muscles, and what effect mutations have on this dynamic behaviour. New research into smooth muscle could benefit from newly developed approaches that could better characterise its organisation.

## Electronic Supplementary Material

Below is the link to the electronic supplementary material.

Supplementary Material 1

## Data Availability

No datasets were generated or analysed during the current study.
