# Supplementary material for: Smooth muscle contractile cytoskeleton in health and disease
Source: J Muscle Res Cell Motil. 2026 Apr 22;47(2):13. doi: 10.1007/s10974-026-09731-4 (PMC13102872; doi:10.1007/s10974-026-09731-4)
Supplement: Supplementary file 1 — Supplementary Material 1 [file 10974_2026_9731_MOESM1_ESM.docx]

**Supplemental Materials (Carrington et al., Smooth muscle contractile cytoskeleton in health and disease)**

**Supplemental Figures: (S1-S3)**


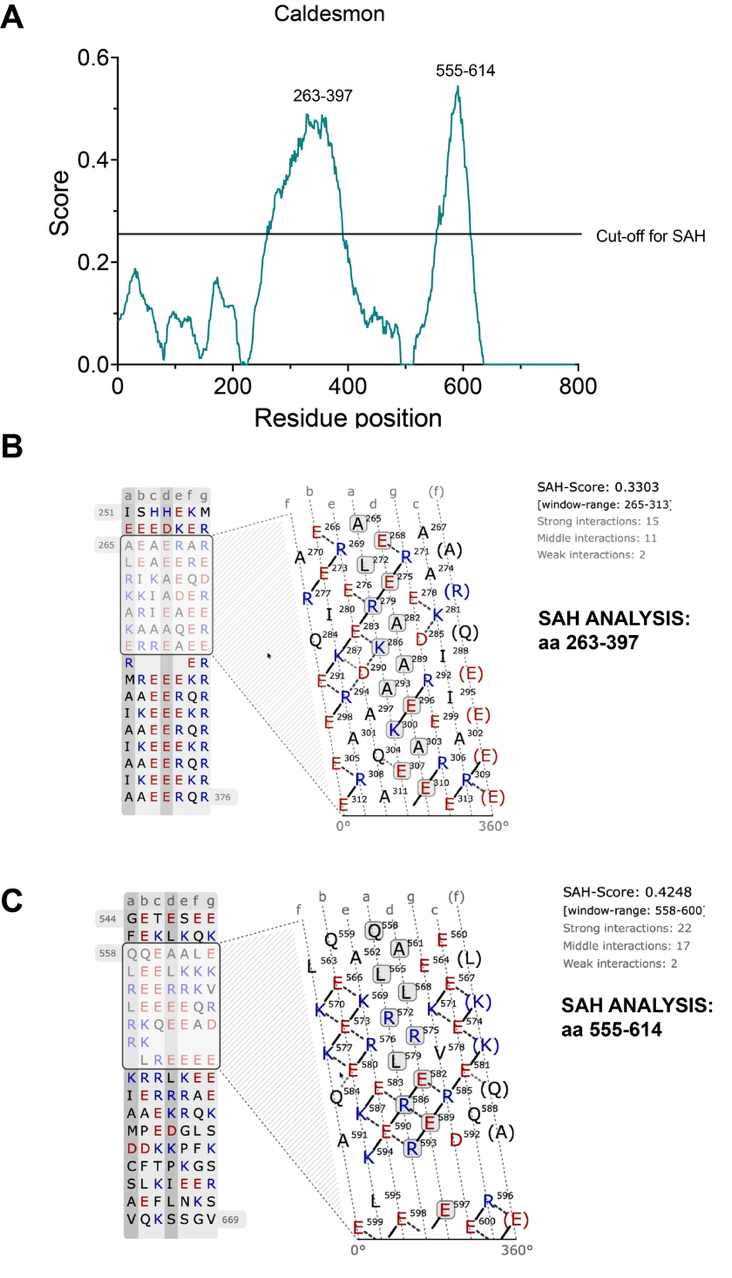


**Fig. S1**: **Prediction of SAH domains in Caldesmon using Waggawagga (Simm et al., 2015). A:** The graphical output: regions above the cutoff are likely SAH domains. **B** and **C**: sequence and heptad net plots for the start of the two regions of SAH: starting at residue 265 and 544 (as output by Waggawagga).


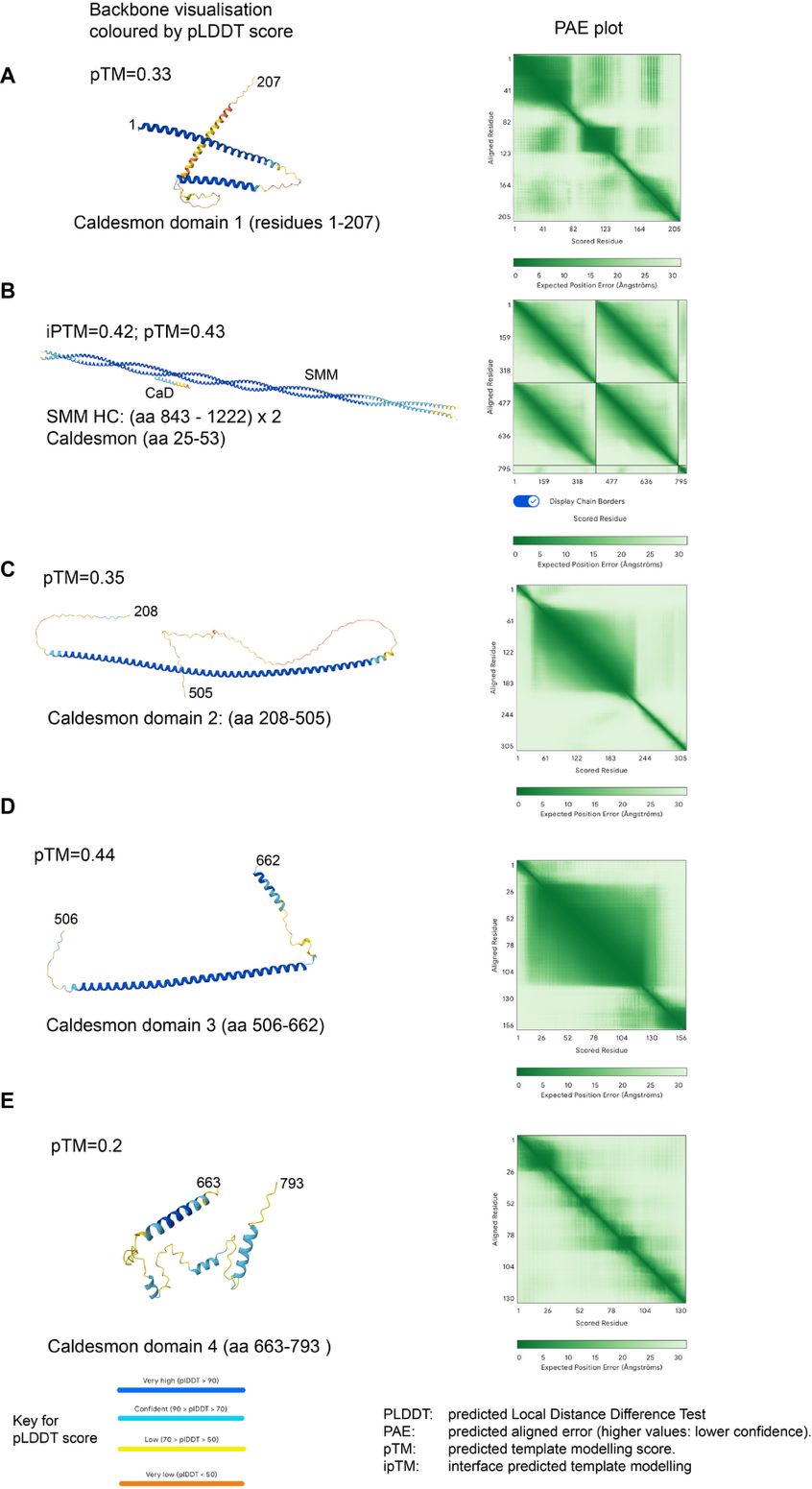


**Fig. S2: Alphafold plots for model structures for the 4 domains of Caldesmon**. Regions coloured blue indicate areas of high confidents (high pLDDT score – see key). Structures shown in Fig. 6. Of particular note, the predicted fold for E has a very low confidence.


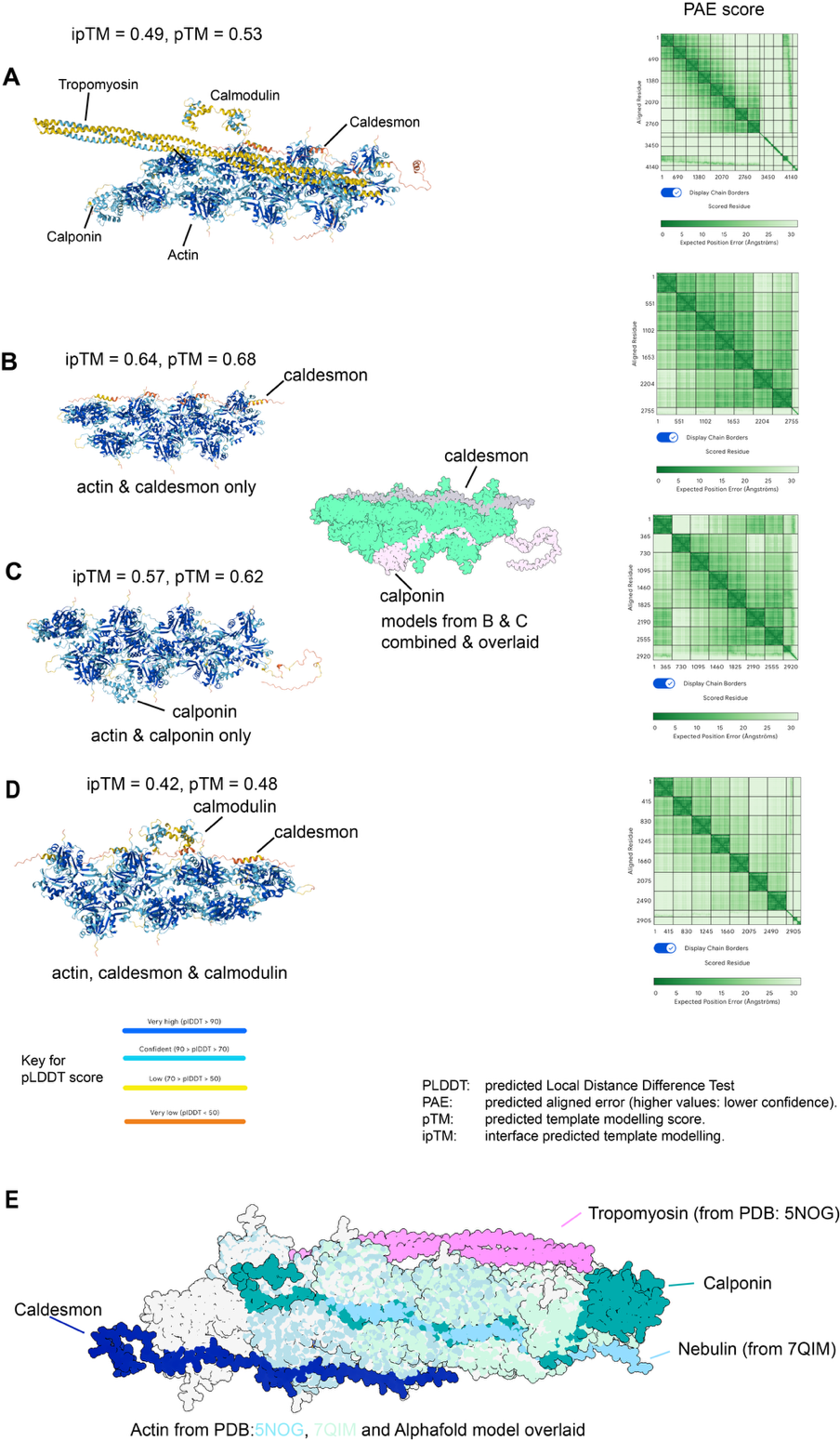


**Fig. S3: Alphafold model for the complex of F-actin, Caldesmon, Calponin, Tropomyosin and Calmodulin**. Calmodulin and tropomyosin structures are poorly predicted in the overall model (**A**), and calmodulin does not seem to interact with the overall structure. To generate the model in **E** (and Fig. 8), the CryoEM structure of tropomyosin on actin (PDB: 5NOG)(Risi et al., 2017) was overlaid on the model structure, and the tropomyosin in the CryoEM structure used to replace that of the Alphafold model, using ChimeraX. The model structure was further overlaid with that of F-actin and nebulin (PDB: 7QIM) (Wang et al., 2022), which shows that Alphafold predicts calponin to run in approximately the same place as nebulin on skeletal muscle actin. **B** and **C** show individual models for actin and caldesmon, and actin and calponin along, with the overlay of these two structures shown on the RHS. D, shows a model for actin, caldesmon (C-term) and calmodulin. The binding site for calmodulin on caldesmon predicted by Alphafold is the strong binding site shown in Fig. 7.

**Supplementary Tables (S1 to S4).**

**Table S1. Mutations in SMM used to generate Fig. 3 (excluding Term mutations).**

| **Mutation** | **Phenotype** | **Reference** | Pubmed ID |
| --- | --- | --- | --- |
| Met1Val | Thoracic aortic aneurysm and dissection | Weerakkody (2018) *Genet Med* 20:1414 | 29543232 |
| Ala2Val | Aortic aneurysm | Lenarduzzi (2023) *Mol Genet Genomic Med* 11: | 36788754 |
| Asp10His | Pancreatic cancer | Fujitani (2023) *Eur J Cancer Prev* 32:286 | 36896836 |
| Glu11Asp | Aortic dissection, acute | Zheng (2018) *Int J Legal Med* 132:1273 | 30056620 |
| Lys54Arg | Thoracic aortic disorder | Overwater (2018) *Hum Mutat* 39:1173 | 29907982 |
| Lys78Glu | Thoracic aortic aneurysms and dissections and patent ductus arteriosus | Harakalova (2013) *Eur J Hum Genet* 21:487 | 22968129 |
| Met90Val | Thoracic aortic aneurysm and dissection | Li (2021) *Eur J Hum Genet* 29:1129 | 33824467 |
| Pro127Ser | Megacystis-microcolon-intestinal hypoperistalsis syndrome | Kloth (2019) *Clin Genet* 96:85 | 31044419 |
| Tyr128Term | Thoracic aortic aneurysm | Ramensky (2021) *Front Genet* 12:709419 | 34691145 |
| Asp140Asn | Aortic aneurysm/dissection in osteogenesis imperfecta | Balasubramanian (2019) *Bone* 121:191 | 30684648 |
| Arg163Trp | Thoracic aortic aneurysm and dissection | Li (2021) *Mol Genet Genomic Med* 9: | 34498425 |
| Thr211Thr | Thoracic aortic aneurysm and dissection | Li (2021) *Mol Genet Genomic Med* 9: | 34498425 |
| Asn230Asn | Developmental disorder | Kaplanis (2020) *Nature* 586:757 | 33057194 |
| Arg247Cys | Altered myosin contractile function | Kuang (2012) *Circ Res* 110:1411 | 22511748 |
| Ile256Val | Patent ductus arteriosus, pulmonary hypoplasia & pulmonary hypertension | Wei (2024) *BMC Med Genomics* 17:135 | 38773466 |
| Asn312Ser | Abdominal aortic aneurysm | van de Luijtgaarden (2015) *Hum Genet* 134:881 | 26017485 |
| Asp323Asn | Aortic aneurysm | Yeung (2017) *Hum Mutat* 38:439 | 28074631 |
| Arg434His | Prostate cancer | Liang (2022) *PLoS Genet* 18:e1010373 | 36095024 |
| Arg439His | Aortic dissection, acute | Zheng (2018) *Int J Legal Med* 132:1273 | 30056620 |
| Ser453Cys | Thoracic aortic disorder | Overwater (2018) *Hum Mutat* 39:1173 | 29907982 |
| Arg501His | Abdominal aortic aneurysm | van de Luijtgaarden (2015) *Hum Genet* 134:881 | 26017485 |
| Glu523Gln | Megacystis-microcolon-intestinal hypoperistalsis syndrome | Kapur (2023) *Pediatr Dev Pathol* 26:39 | 36571289 |
| Arg524Term | Megacystis-microcolon-intestinal hypoperistalsis syndrome | Billon (2020) *Clin Genet* 98:261 | 32621347 |
| Asp629Gly | Aortic dissection | Fang (2017) *Sci Rep* 7:10035 | 28855619 |
| Arg651Cys | Thoracic aortic aneurysm and dissection | Arnaud (2019) *Genet Med* 21:2015 | 30739908 |
| Arg669Cys | Thoracic aortic aneurysm and dissection | Kathiravel (2013) *Mol Cell Probes* 27:103 | 23142374 |
| Arg677His | Megacystis-microcolon-intestinal hypoperistalsis syndrome | Wang (2019) *J Hum Genet* 64:1067 | 31427716 |
| Arg700Trp | Chronic intestinal pseudo-obstruction | Kapur (2023) *Pediatr Dev Pathol* 26:39 | 36571289 |
| Arg712Gln | Thoracic aortic aneurysm and patent ductus arteriosus | Pannu (2007) *Hum Mol Genet* 16:3453 | 17666408 |
| Gly714Val | Megacystis-microcolon-intestinal hypoperistalsis syndrome | Kapur (2023) *Pediatr Dev Pathol* 26:39 | 36571289 |
| Arg718Trp | Thoracic aortic aneurysm and dissection | Arnaud (2019) *Genet Med* 21:2015 | 30739908 |
| Ala732Val | Marfan syndrome with aortopathy | Wooderchak-Donahue (2015) *Am J Med Genet A* 167:1747 | 25944730 |
| Pro758Thr | Thoracic aortic aneurysm and dissection | Li (2021) *Mol Genet Genomic Med* 9: | 34498425 |
| Ser766Asn | Aortic dissection | Stranneheim (2021) *Genome Med* 13:40 | 33726816 |
| Thr814Ala | Cardiomyopathy, non-ischemic | Vokač (2024) *Genes (Basel)* 15:72 | 38254962 |
| Trp832Cys | Marfan syndrome with aortopathy | Wooderchak-Donahue (2015) *Am J Med Genet A* 167:1747 | 25944730 |
| Glu866Lys | Thoracic aortic aneurysm and dissection | Arnaud (2019) *Genet Med* 21:2015 | 30739908 |
| Thr886Thr | Autism | Zhou (2022) *Nat Genet* 54:1305 | 35982159 |
| Asn890Asn | Autism spectrum disorder | Fu (2022) *Nat Genet* 54:1320 | 35982160 |
| His925Tyr | Unilateral microphthalmia, isolated | Li (2022) *Am J Med Genet A* 188:2376 | 35716026 |
| Glu934Glu | Autism spectrum disorder | Krupp (2017) *Am J Hum Genet* 101:369 | 28867142 |
| Glu984Asp | Aortic dissection, acute | Zheng (2018) *Int J Legal Med* 132:1273 | 30056620 |
| Ser1049Arg | Thoracic aortic aneurysm and dissection | Li (2021) *Mol Genet Genomic Med* 9: | 34498425 |
| Ile1071Met | Thoracic aortic aneurysm | Keravnou (2018) *BMC Med Genet* 19:208 | 30526509 |
| Lys1082Glu | Thoracic aortic aneurysm and dissection | Li (2021) *Mol Genet Genomic Med* 9: | 34498425 |
| Glu1125Asp | Thoracic aortic aneurysm and dissection | Li (2021) *Eur J Hum Genet* 29:1129 | 33824467 |
| Glu1130Lys | Schizophrenia | Fromer (2014) *Nature* 506:179 | 24463507 |
| Arg1131Trp | Thoracic aortic aneurysm and dissection | Li (2021) *Eur J Hum Genet* 29:1129 | 33824467 |
| Lys1141Gln | Chronic kidney disease & hypertension | Geraghty (2023) *J Rare Dis (Berlin)* 2:9 | 37288276 |
| Arg1142Term | Megacystis, Bilateral hydronephrosis, Absent stomach, polyhydramnios, Microcolon, Intestinal hypoperistalsis | Monies (2019) *Am J Hum Genet* 104:1182 | 31130284 |
| Arg1188Trp | Thoracic aortic disorder | Overwater (2018) *Hum Mutat* 39:1173 | 29907982 |
| Lys1200Term | Megacystis-microcolon-intestinal hypoperistalsis syndrome | Gauthier (2015) *Eur J Hum Genet* 23:1266 | 25407000 |
| His1201Gln | Sudden infant death syndrome | Neubauer (2017) *Eur J Hum Genet* 25:404 | 28074886 |
| Glu1206Lys | Patent foramen ovale | Li (2024) *Front Genet* 15: | 38808331 |
| Ala1234Thr | Thoracic aortic aneurysm | Proost (2015) *Hum Mutat* 36:808 | 25907466 |
| Leu1243Pro | Thoracic aortic aneurysm and dissection | Li (2021) *Mol Genet Genomic Med* 9: | 34498425 |
| Lys1256Gln | Thoracic aortic aneurysm and dissection | Weerakkody (2018) *Genet Med* 20:1414 | 29543232 |
| Leu1264Pro | Thoracic aortic aneurysm and patent ductus arteriosus | Pannu (2007) *Hum Mol Genet* 16:3453 | 17666408 |
| Asn1279Asn | Autism spectrum disorder | Fu (2022) *Nat Genet* 54:1320 | 35982160 |
| Gln1286Term | Marfan syndrome with aortopathy | Wooderchak-Donahue (2015) *Am J Med Genet A* 167:1747 | 25944730 |
| Glu1290Gln | Patent arterial duct | Zhu (2007) *Cardiol Young* 17:666 | 17956658 |
| Ala1412Val | Marfan syndrome with aortopathy | Wooderchak-Donahue (2015) *Am J Med Genet A* 167:1747 | 25944730 |
| Lys1449Gln | Ductus arteriosus aneurysm | Ardhanari (2020) *Cardiol Young* 30:123 | 31916526 |
| Ala1468Val | Thoracic aortic aneurysm and dissection | Li (2021) *Mol Genet Genomic Med* 9: | 34498425 |
| Lys1481Met | Aortic dissection, Stanford type A | Chen (2021) *J Thorac Dis* 13:4008 | 34422331 |
| Arg1504Gly | Thoracic aortic aneurysm and dissection | Arnaud (2019) *Genet Med* 21:2015 | 30739908 |
| Arg1535Gln | Aortic aneurysm | Sakai (2012) *Hum Genet* 131:591 | 22001912 |
| Thr1546Met | Thoracic aortic aneurysm | Pucci (2019) *Ann Thorac Surg* :S0003 | 31473177 |
| Thr1558Met | Abdominal aortic aneurysm | van de Luijtgaarden (2015) *Hum Genet* 134:881 | 26017485 |
| Lys1573Glu | Sudden unexpected death | Hata (2017) *Clin Med Insights Case Rep* 10:1179547617702884 | 28469501 |
| Asp1579Asn | Thoracic aortic disorder | Overwater (2018) *Hum Mutat* 39:1173 | 29907982 |
| Arg1583Gln | Thoracic aortic aneurysm | Bee (2012) *Circ Cardiovasc Genet* 5:621 | 23099432 |
| Arg1583Trp | Thoracic aortic aneurysm and dissection | Weerakkody (2018) *Genet Med* 20:1414 | 29543232 |
| Ala1615Thr | Aortic dissection, Stanford type A | Chen (2021) *J Thorac Dis* 13:4008 | 34422331 |
| Ala1617Ser | Thoracic aortic disorder | Overwater (2018) *Hum Mutat* 39:1173 | 29907982 |
| Lys1621Gln | Thoracic aortic disorder | Overwater (2018) *Hum Mutat* 39:1173 | 29907982 |
| Ser1635Thr | Intracranial aneurysm | Kurtelius (2019) *J Am Heart Assoc* 8:e013277 | 31538843 |
| Arg1648Cys | Aortic aneurysm | Sakai (2012) *Hum Genet* 131:591 | 22001912 |
| Glu1694Gly | Aortic dissection | Zeng (2022) *Front Cardiovasc Med* 9:1030160 | 36440024 |
| Ala1698Thr | Thoracic aortic aneurysm and dissection | Li (2021) *Mol Genet Genomic Med* 9: | 34498425 |
| Glu1747Gln | Aortic aneurysm | Seo (2020) *Clin Genet* 98:562 | 32901917 |
| Gln1749Pro | Thoracic aortic disorder | Overwater (2018) *Hum Mutat* 39:1173 | 29907982 |
| Arg1758Gln | Aortic aneurysm, familial thoracic 4 | Zhu (2006) *Nat Genet* 38:343 | 16444274 |
| Arg1758Trp | Thoracic aortic disorder | Overwater (2018) *Hum Mutat* 39:1173 | 29907982 |
| Val1759Val | Autism | Zhou (2022) *Nat Genet* 54:1305 | 35982159 |
| Leu1801Leu | Aortopathy | Renner (2019) *Genet Med* 21:1832 | 30675029 |
| Glu1833Asp | Thoracic aortic aneurysm and dissection | Poninska (2016) *J Transl Med* 14:115 | 27146836 |
| Arg1862His | Thoracic aortic aneurysm and dissection | Li (2021) *Eur J Hum Genet* 29:1129 | 33824467 |
| Lys1863Arg | Thoracic aortic aneurysm and dissection | Li (2021) *Mol Genet Genomic Med* 9: | 34498425 |
| Glu1892Asp | Abdominal aortic aneurysm | van de Luijtgaarden (2015) *Hum Genet* 134:881 | 26017485 |
| Arg1895Cys | Thoracic aortic aneurysm and dissection | Li (2021) *Eur J Hum Genet* 29:1129 | 33824467 |
| Asn1899Ser | Marfan syndrome | Lin (2022) *J Pers Med* 12:198 | 35207686 |
| Thr1911Met | Thoracic aortic aneurysm and dissection | Li (2021) *Eur J Hum Genet* 29:1129 | 33824467 |
| Arg1930Term | Congenital heart disease | Morton (2021) *JAMA Cardiol* 6:457 | 33084842 |
| Thr1934Ser | Congenital heart disease | Izarzugaza (2020) *Genome Med* 12:76 | 32859249 |

**Table S2. Mutations in ACTA2 (smooth muscle actin) used to generate Fig. 4 (excluding Term mutations).**

| **HGVS** | **Phenotype** | **Reference** | Pubmed ID |
| --- | --- | --- | --- |
| Glu5Gln | Thoracic aortic disorder | Overwater (2018) *Hum Mutat* 39:1173 | 29907982 |
| Ser16Pro | Thoracic aortic aneurysm and dissection | van den Bersselaar (2022) *Genet Med* 24:2112 | 36053285 |
| Cys19Arg | Aortic disease | Regalado (2015) *Circ Cardiovasc Genet* 8:457 | 25759435 |
| Gly22Ser | Aortic disease | Regalado (2015) *Circ Cardiovasc Genet* 8:457 | 25759435 |
| Asp26Tyr | Thoracic aortic aneurysm and dissection | Yoo (2010) *Ann Clin Lab Sci* 40:278 | 20689142 |
| Asp27Asn | Thoracic aortic aneurysm and dissection | Yang (2023) *J Thorac Cardiovasc Surg* 166:1594 | 36517271 |
| Asp27Gly | Thoracic aortic aneurysm and dissection | Poninska (2016) *J Transl Med* 14:115 | 27146836 |
| Pro34Arg | Thoracic aortic aneurysm and dissection | van den Bersselaar (2022) *Genet Med* 24:2112 | 36053285 |
| Gly38Arg | Thoracic aortic aneurysm and dissection | Renard (2013) *Int J Cardiol* 165:314 | 21937134 |
| Arg39Cys | Thoracic aortic aneurysm and dissection | Hoffjan (2011) *Eur J Hum Genet* 19:520 | 21248741 |
| Arg39Gly | Aortic disease | Regalado (2015) *Circ Cardiovasc Genet* 8:457 | 25759435 |
| Arg39His | Thoracic aortic disease, coronary artery disease & strokes | Guo (2009) *Am J Hum Genet* 84:617 | 19409525 |
| Arg39Ser | Aortic dissection, acute | Zheng (2018) *Int J Legal Med* 132:1273 | 30056620 |
| Pro40Ser | Thoracic aortic aneurysm and dissection | Proost (2015) *Hum Mutat* 36:808 | 25907466 |
| His42Asn | Thoracic aortic aneurysm and dissection | Renard (2013) *Int J Cardiol* 165:314 | 21937134 |
| Val45Leu | Thoracic aortic aneurysm and dissection | Disabella (2011) *Heart* 97:321 | 21212136 |
| Met46Arg | Patent ductus arteriosus | Zhang (2019) *Can J Neurol Sci* :1 | 30975232 |
| Gly48Val | Aortic disease | Regalado (2015) *Circ Cardiovasc Genet* 8:457 | 25759435 |
| Met49Val | Thoracic aortic aneurysm and dissection | Hoffjan (2011) *Eur J Hum Genet* 19:520 | 21248741 |
| Asp58Asn | Aortic dissection, acute | Zheng (2018) *Int J Legal Med* 132:1273 | 30056620 |
| Ala60Glu | Thoracic aortic disorder | Overwater (2018) *Hum Mutat* 39:1173 | 29907982 |
| Gln61Arg | Thoracic aortic aneurysm and dissection | Renard (2013) *Int J Cardiol* 165:314 | 21937134 |
| Arg64Lys | Aortic disease | Regalado (2015) *Circ Cardiovasc Genet* 8:457 | 25759435 |
| Ile66Asn | Multisystem smooth muscle dysfunction | Kaw (2022) *Am J Med Genet A* 188:2389 | 35567597 |
| Leu69Gln | Aortic disease | Regalado (2015) *Circ Cardiovasc Genet* 8:457 | 25759435 |
| Pro72Gln | Thoracic aortic aneurysm and dissection | Guo (2009) *Am J Hum Genet* 84:617 | 19409525 |
| Asp82Glu | Thoracic aortic aneurysm and dissection | Disabella (2011) *Heart* 97:321 | 21212136 |
| Met84Val | Thoracic aortic aneurysm and dissection | Roque Rodríguez (2019) *Rev Esp Cardiol (Engl Ed)* 73:334 | 31879232 |
| Ile87Met | Thoracic aortic aneurysm and dissection | Yang (2023) *J Thorac Cardiovasc Surg* 166:1594 | 36517271 |
| Trp88Arg | Aortic disease | Regalado (2015) *Circ Cardiovasc Genet* 8:457 | 25759435 |
| Glu95Lys | Thoracic aortic aneurysm | Strecker (2022) *Virchows Arch* epub:epub | 35896809 |
| Ala99Gly | Thoracic aortic aneurysm and dissection | van den Bersselaar (2022) *Genet Med* 24:2112 | 36053285 |
| Thr108Met | Thoracic aortic aneurysm | Bee (2012) *Circ Cardiovasc Genet* 5:621 | 23099432 |
| Asn113Thr | Thoracic aortic aneurysm and dissection | Lerner-Ellis (2014) *Mol Genet Metab* 112:171 | 24793577 |
| Pro114Ser | Developmental disorder | Kaplanis (2020) *Nature* 586:757 | 33057194 |
| Asn117Ile | Thoracic aortic aneurysm and dissection | Ke (2016) *BMC Med Genet* 17:45 | 27431987 |
| Asn117Lys | Cerebral arteriopathy and mydriasis | Mc Glacken-Byrne (2020) *BMC Ophthalmol* 20:68 | 32093627 |
| Asn117Ser | Thoracic aortic aneurysm and dissection | Poninska (2016) *J Transl Med* 14:115 | 27146836 |
| Asn117Thr | Thoracic aortic aneurysm and dissection | Guo (2007) *Nat Genet* 39:1488 | 17994018 |
| Arg118Gln | Thoracic aortic aneurysm and dissection | Guo (2007) *Nat Genet* 39:1488 | 17994018 |
| Met134Thr | Aortic dissection | Fang (2017) *Sci Rep* 7:10035 | 28855619 |
| Tyr135His | Thoracic aortic aneurysm and dissection | Guo (2007) *Nat Genet* 39:1488 | 17994018 |
| Ala140Ala | Aortopathy | Renner (2019) *Genet Med* 21:1832 | 30675029 |
| Ala140Val | Thoracic aortic aneurysm and dissection | Lerner-Ellis (2014) *Mol Genet Metab* 112:171 | 24793577 |
| Val141Ala | Aortic disease | Regalado (2015) *Circ Cardiovasc Genet* 8:457 | 25759435 |
| Tyr145Cys | Thoracic aortic aneurysm and dissection | Morisaki (2009) *Hum Mutat* 30:1406 | 19639654 |
| Gly148Arg | Aortic disease | Regalado (2015) *Circ Cardiovasc Genet* 8:457 | 25759435 |
| Arg149Cys | Thoracic aortic aneurysm and dissection | Guo (2007) *Nat Genet* 39:1488 | 17994018 |
| Arg149His | Aortic dissection, Stanford type A | Chen (2021) *J Thorac Dis* 13:4008 | 34422331 |
| Val154Ala | Thoracic aortic aneurysm and dissection | Guo (2007) *Nat Genet* 39:1488 | 17994018 |
| Val154Met | Thoracic aortic aneurysm and dissection | Guo (2022) *Front Genet* 13: | 35754816 |
| Gly160Asp | Thoracic aortic aneurysm and dissection | Guo (2009) *Am J Hum Genet* 84:617 | 19409525 |
| Val161Ala | Aortic aneurysm | Sakai (2012) *Hum Genet* 131:591 | 22001912 |
| Thr162Asn | Thoracic aortic aneurysm and dissection | van den Bersselaar (2022) *Genet Med* 24:2112 | 36053285 |
| His163Gln | Aortic disease | Regalado (2015) *Circ Cardiovasc Genet* 8:457 | 25759435 |
| Pro166Thr | Aortic disease | Regalado (2015) *Circ Cardiovasc Genet* 8:457 | 25759435 |
| Tyr168Asn | Thoracic aortic aneurysm and dissection | Ke (2016) *BMC Med Genet* 17:45 | 27431987 |
| Ala172Val | Developmental disorder | Kaplanis (2020) *Nature* 586:757 | 33057194 |
| His175Asp | Thoracic aortic aneurysm and dissection | van den Bersselaar (2022) *Genet Med* 24:2112 | 36053285 |
| Ala176Thr | Thoracic aortic aneurysm and dissection | van den Bersselaar (2022) *Genet Med* 24:2112 | 36053285 |
| Arg179Cys | Cardiovascular, autonomic & brain anomalies | Meuwissen (2013) *Am J Med Genet A* 161:1376 | 23613326 |
| Arg179Gly | Multisystem smooth muscle dysfunction | Kaw (2022) *Am J Med Genet A* 188:2389 | 35567597 |
| Arg179His | Multisystem smooth muscle dysfunction | Milewicz (2010) *Am J Med Genet A* 152A:2437 | 20734336 |
| Arg179Leu | Cerebrovascular disease | Munot (2012) *Brain* 135:2506 | 22831780 |
| Arg179Ser | Multisystem smooth muscle dysfunction | Regalado (2018) *Genet Med* 20:1206 | 29300374 |
| Asp181Val | Multisystem smooth muscle dysfunction | Kaw (2022) *Am J Med Genet A* 188:2389 | 35567597 |
| Arg185Gln | Thoracic aortic disease & coronary artery disease | Guo (2009) *Am J Hum Genet* 84:617 | 19409525 |
| Tyr190Ser | Marfan syndrome with aortopathy | Wooderchak-Donahue (2015) *Am J Med Genet A* 167:1747 | 25944730 |
| Met192Ile | Thoracic aortic aneurysm and dissection | Li (2021) *Eur J Hum Genet* 29:1129 | 33824467 |
| Met192Val | Aortic dissection | Fang (2017) *Sci Rep* 7:10035 | 28855619 |
| Thr196Asn | Thoracic aortic aneurysm and dissection | van den Bersselaar (2022) *Genet Med* 24:2112 | 36053285 |
| Arg198Cys | Aortic disease | Regalado (2015) *Circ Cardiovasc Genet* 8:457 | 25759435 |
| Arg198His | Aortic disease | Regalado (2015) *Circ Cardiovasc Genet* 8:457 | 25759435 |
| Val203Ile | Thoracic aortic aneurysm and dissection | Lerner-Ellis (2014) *Mol Genet Metab* 112:171 | 24793577 |
| Val203Leu | Thoracic aortic disorder | Overwater (2018) *Hum Mutat* 39:1173 | 29907982 |
| Thr204Ile | Multisystem smooth muscle dysfunction | Kaw (2022) *Am J Med Genet A* 188:2389 | 35567597 |
| Arg208Cys | Thoracic aortic aneurysm and dissection | Yang (2023) *J Thorac Cardiovasc Surg* 166:1594 | 36517271 |
| Arg208His | Thoracic aortic aneurysm and dissection | van den Bersselaar (2022) *Genet Med* 24:2112 | 36053285 |
| Arg212Gln | Thoracic aortic disease, coronary artery disease & strokes | Guo (2009) *Am J Hum Genet* 84:617 | 19409525 |
| Glu216Lys | Thoracic aortic aneurysm and dissection | Yang (2023) *J Thorac Cardiovasc Surg* 166:1594 | 36517271 |
| Glu243Lys | Thoracic aortic aneurysm and dissection | Disabella (2011) *Heart* 97:321 | 21212136 |
| Leu244Phe | Aortic disease | Regalado (2015) *Circ Cardiovasc Genet* 8:457 | 25759435 |
| Pro245His | Thoracic aortic disease & strokes | Guo (2009) *Am J Hum Genet* 84:617 | 19409525 |
| Pro245Leu | Aortic disease | Regalado (2015) *Circ Cardiovasc Genet* 8:457 | 25759435 |
| Asp246Val | Thoracic aortic dissection | Chen (2023) *Gene* 887: | 37625564 |
| Ile250Leu | Thoracic aortic disease & strokes | Guo (2009) *Am J Hum Genet* 84:617 | 19409525 |
| Gly253Arg | Thoracic aortic aneurysm and dissection | Yang (2023) *J Thorac Cardiovasc Surg* 166:1594 | 36517271 |
| Arg256His | Thoracic aortic aneurysm and dissection | Weerakkody (2018) *Genet Med* 20:1414 | 29543232 |
| Arg256Ser | Thoracic aortic aneurysm and dissection | Stranneheim (2021) *Genome Med* 13:40 | 33726816 |
| Arg258Cys | Thoracic aortic aneurysm and dissection | Guo (2007) *Nat Genet* 39:1488 | 17994018 |
| Arg258His | Thoracic aortic disease & strokes | Guo (2009) *Am J Hum Genet* 84:617 | 19409525 |
| Gly270Arg | Aortic disease | Regalado (2015) *Circ Cardiovasc Genet* 8:457 | 25759435 |
| Gly270Glu | Thoracic aortic aneurysm | Bee (2012) *Circ Cardiovasc Genet* 5:621 | 23099432 |
| Glu272Gly | Thoracic aortic aneurysm and dissection | Li (2021) *Eur J Hum Genet* 29:1129 | 33824467 |
| Gly275Ala | Thoracic aortic aneurysm and dissection | Regalado (2014) *Am J Med Genet A* 164:106 | 24243736 |
| Thr279Ala | Thoracic aortic disorder | Overwater (2018) *Hum Mutat* 39:1173 | 29907982 |
| Asn282Tyr | Thoracic aortic aneurysm and dissection | Li (2021) *Mol Genet Genomic Med* 9: | 34498425 |
| Ile284Asn | Thoracic aortic aneurysm and dissection | Weerakkody (2018) *Genet Med* 20:1414 | 29543232 |
| Met285Thr | Thoracic aortic disorder | Overwater (2018) *Hum Mutat* 39:1173 | 29907982 |
| Arg292Gly | Thoracic aortic aneurysm and dissection | Guo (2007) *Nat Genet* 39:1488 | 17994018 |
| Asn298Ser | Moyamoya disease | Chang (2022) *Cell Mol Neurobiol* : | 36580209 |
| Ser302Ala | Thoracic aortic aneurysm and dissection | Regalado (2014) *Am J Med Genet A* 164:106 | 24243736 |
| Gly304Arg | Thoracic aortic aneurysm and dissection | Hoffjan (2011) *Eur J Hum Genet* 19:520 | 21248741 |
| Gly304Ser | Thoracic aortic disorder, nonsyndromic | Campens (2015) *Orphanet J Rare Dis* 10:9 | 25644172 |
| Arg314Gln | Thoracic aortic aneurysm and dissection | Yang (2023) *J Thorac Cardiovasc Surg* 166:1594 | 36517271 |
| Arg314Term | Thoracic aortic aneurysm and dissection | Renard (2013) *Int J Cardiol* 165:314 | 21937134 |
| Glu318Lys | Thoracic aortic aneurysm and dissection | Arnaud (2019) *Genet Med* 21:2015 | 30739908 |
| Thr326Asn | Thoracic aortic disease, coronary artery disease & strokes | Guo (2009) *Am J Hum Genet* 84:617 | 19409525 |
| Lys328Asn | Thoracic aortic aneurysm and dissection | Ware (2014) *Pediatrics* 134:e1218 | 25225139 |
| Pro335Arg | Aortic dissection | Delsart (2021) *Ann Vasc Surg* 77:348.e7 | 34437965 |
| Gly344Ser | Aortic dissection, acute | Zheng (2018) *Int J Legal Med* 132:1273 | 30056620 |
| Leu348Arg | Thoracic aortic aneurysm and dissection | Ke (2016) *BMC Med Genet* 17:45 | 27431987 |
| Thr353Asn | Thoracic aortic aneurysm and dissection | Guo (2007) *Nat Genet* 39:1488 | 17994018 |
| Gly368Arg | Schizophrenia | Fromer (2014) *Nature* 506:179 | 24463507 |
| Arg374Cys | Thoracic aortic disorder | Overwater (2018) *Hum Mutat* 39:1173 | 29907982 |

**Table S3. Mutations in ACTG2 used to generate Fig. 4.**

| **HGVS** | **Phenotype** | **Reference** | Pubmed ID |
| --- | --- | --- | --- |
| Val10Met | Visceral myopathy, familial | Mori (2022) *JPGN Rep* 3:e258 | 37168481 |
| Ala23Thr | Intestinal dysmotility | Sandy (2022) *J Pediatr Gastroenterol Nutr* 74:575 | 35149643 |
| Phe32Phe | Developmental disorder | Kaplanis (2020) *Nature* 586:757 | 33057194 |
| Arg38His | Chronic intestinal pseudo-obstruction | Matera (2016) *Eur J Hum Genet* 24:1211 | 26813947 |
| Pro39Arg | Chronic intestinal pseudo-obstruction | Lee (2019) *J Pediatr Gastroenterol Nutr* 68:e16 | 30334933 |
| Pro39Leu | Visceral myopathy, familial | Assia Batzir (2020) *Hum Mutat* 41:641 | 31769566 |
| Arg40Cys | Megacystis-microcolon-intestinal hypoperistalsis syndrome | Wangler (2014) *PLoS Genet* 10:1004258 | 24676022 |
| Arg40His | Megacystis-microcolon-intestinal hypoperistalsis syndrome | Wangler (2014) *PLoS Genet* 10:1004258 | 24676022 |
| His41Gln | Chronic intestinal pseudo-obstruction | Matera (2021) *Clin Genet* 99:430 | 33294969 |
| Val44Ala | Visceral myopathy, familial | Assia Batzir (2020) *Hum Mutat* 41:641 | 31769566 |
| Met45Thr | Megacystis-microcolon-intestinal hypoperistalsis syndrome | Wangler (2014) *PLoS Genet* 10:1004258 | 24676022 |
| Ser61Arg | Intestinal dysmotility | Sandy (2022) *J Pediatr Gastroenterol Nutr* 74:575 | 35149643 |
| Arg63Gln | Megacystis-microcolon-intestinal hypoperistalsis syndrome | Halim (2016) *Hum Mol Genet* 25:571 | 26647307 |
| Arg63Gly | Megacystis-microcolon-intestinal hypoperistalsis syndrome | Wangler (2014) *PLoS Genet* 10:1004258 | 24676022 |
| Glu73Gln | Intestinal dysmotility | Sandy (2022) *J Pediatr Gastroenterol Nutr* 74:575 | 35149643 |
| Ile76Phe | Visceral myopathy | Geraghty (2023) *J Rare Dis (Berlin)* 2:9 | 37288276 |
| Arg96His | Kidney and urinary tract abnormalities (CAKUT) | Geraghty (2023) *J Rare Dis (Berlin)* 2:9 | 37288276 |
| Pro113Ala | Intestinal dysmotility | Sandy (2022) *J Pediatr Gastroenterol Nutr* 74:575 | 35149643 |
| Pro113Leu | Visceral myopathy | Geraghty (2023) *J Rare Dis (Berlin)* 2:9 | 37288276 |
| Pro113Ser | Intestinal pseudo-obstruction | Wei (2021) *J Pediatr Gastroenterol Nutr* 72:36 | 32810037 |
| Asn116Lys | Intestinal dysmotility | Sandy (2022) *J Pediatr Gastroenterol Nutr* 74:575 | 35149643 |
| Asn116Ser | Intestinal dysmotility | Sandy (2022) *J Pediatr Gastroenterol Nutr* 74:575 | 35149643 |
| Lys119Arg | Visceral leiomyopathy, degenerative | Maluleke (2019) *Pediatr Surg Int* 35:439 | 30430282 |
| Met120Thr | Visceral myopathy | Kapur (2022) *Pediatr Dev Pathol* 25:581 | 35695198 |
| Asn129Ser | Kidney disease & recurrent urinary tract infections | Geraghty (2023) *J Rare Dis (Berlin)* 2:9 | 37288276 |
| Tyr134Asn | Megacystis-microcolon-intestinal hypoperistalsis syndrome | Wangler (2014) *PLoS Genet* 10:1004258 | 24676022 |
| Leu143Phe | Visceral myopathy, familial | Assia Batzir (2020) *Hum Mutat* 41:641 | 31769566 |
| Gly147Arg | Megacystis-microcolon-intestinal hypoperistalsis syndrome | Kapur (2022) *Pediatr Dev Pathol* 25:581 | 35695198 |
| Gly147Cys | Chronic intestinal pseudo-obstruction | Collins (2018) *Int J Surg Pathol* :1066896918786586 | 30019982 |
| Arg148Cys | Visceral myopathy | Geraghty (2023) *J Rare Dis (Berlin)* 2:9 | 37288276 |
| Arg148Leu | Chronic intestinal pseudo-obstruction | Ravenscroft (2018) *Neurogastroenterol Motil* 30:e13371 | 29781137 |
| Arg148Ser | Visceral myopathy, familial | Lehtonen (2012) *Gastroenterology* 143:1482 | 22960657 |
| Thr149Arg | Chronic intestinal pseudo-obstruction | Matera (2021) *Clin Genet* 99:430 | 33294969 |
| Val160Ile | Intestinal dysmotility | Sandy (2022) *J Pediatr Gastroenterol Nutr* 74:575 | 35149643 |
| Arg178Cys | Megacystis-microcolon-intestinal hypoperistalsis syndrome | Thorson (2014) *Hum Genet* 133:737 | 24337657 |
| Arg178His | Megacystis-microcolon-intestinal hypoperistalsis syndrome | Wangler (2014) *PLoS Genet* 10:1004258 | 24676022 |
| Arg178Leu | Megacystis-microcolon-intestinal hypoperistalsis syndrome | Thorson (2014) *Hum Genet* 133:737 | 24337657 |
| Arg178Ser | Megacystis-microcolon-intestinal hypoperistalsis syndrome | Korğalı (2018) *Fetal Pediatr Pathol* 37:109 | 29608093 |
| Asp185Tyr | Intestinal pseudo-obstruction | Wei (2021) *J Pediatr Gastroenterol Nutr* 72:36 | 32810037 |
| Tyr189His | Intestinal dysmotility | Sandy (2022) *J Pediatr Gastroenterol Nutr* 74:575 | 35149643 |
| Ile193Phe | Chronic intestinal pseudo-obstruction | Hahn (2022) *J Neurogastroenterol Motil* 28:104 | 34980693 |
| Thr195Ile | Chronic intestinal pseudo-obstruction | Moreno (2016) *Am J Med Genet A* 170:2965 | 27481187 |
| Glu196Asp | Chronic intestinal pseudo-obstruction | Matera (2021) *Clin Genet* 99:430 | 33294969 |
| Glu196Asp | Developmental disorder | Kaplanis (2020) *Nature* 586:757 | 33057194 |
| Gly198Asp | Megacystis-microcolon-intestinal hypoperistalsis syndrome | Wangler (2014) *PLoS Genet* 10:1004258 | 24676022 |
| Ala205Thr | Microcolon-megacystic syndrome | Stark (2016) *Genet Med* 18:1090 | 26938784 |
| Glu206Val | Megacystis-microcolon-intestinal hypoperistalsis syndrome | Sandy (2022) *J Pediatr Gastroenterol Nutr* 74:575 | 35149643 |
| Arg211Gln | Visceral myopathy, familial | Whittington (2017) *Case Rep Genet* 2017:9146507 | 29387497 |
| Arg211Term | Severe intestinal pseudo-obstruction | Monies (2017) *Genet Med* 19:1144 | 28383543 |
| Asp245Gly | Intestinal pseudo-obstruction | Wei (2021) *J Pediatr Gastroenterol Nutr* 72:36 | 32810037 |
| Arg257Cys | Megacystis-microcolon-intestinal hypoperistalsis syndrome | Wangler (2014) *PLoS Genet* 10:1004258 | 24676022 |
| Arg257His | Visceral myopathy, familial | Iglesias (2014) *Genet Med* 16:922 | 24901346 |
| Met284Val | Prune belly syndrome | Geraghty (2023) *J Rare Dis (Berlin)* 2:9 | 37288276 |
| Asp289Gly | Kidney and urinary tract abnormalities (CAKUT) | Liu (2022) *Nephrol Dial Transplant* : | 36549658 |
| Pro323Leu | Intestinal pseudo-obstruction | Sandy (2022) *J Pediatr Gastroenterol Nutr* 74:575 | 35149643 |
| Arg336Trp | Chronic intestinal pseudo-obstruction | Matera (2021) *Clin Genet* 99:430 | 33294969 |
| Ser345Cys | Intestinal dysmotility | Sandy (2022) *J Pediatr Gastroenterol Nutr* 74:575 | 35149643 |

**Table S4. Mutations in TPM1 used to generate Fig. 5.** Shared exons with TPM 1.3 and 1.4 found in smooth muscle are indicated. Term mutations are excluded.

| EXON | Mutation | **Reported phenotype** | **Reference** |
| --- | --- | --- | --- |
| 1a  (Shared) | Met8Arg | Cardiomyopathy, dilated | (Lakdawala et al., 2012; Racca et al., 2020; Walsh et al., 2017) |
|  | Met10Leu | Cardiomyopathy, dilated | (Hu et al., 2018) |
|  | Asp14Gly | Noncompaction, left ventricular | (Hirono et al., 2020; Wang et al., 2017) |
|  | Asp14His | Cardiomyopathy, dilated | (Hu et al., 2018) |
|  | Lys15Asn | Cardiomyopathy, dilated | (Colpan et al., 2017; Hu et al., 2018) |
|  | Glu16Gln | Cardiomyopathy, hypertrophic | (Gomez et al., 2014; Walsh et al., 2017) |
|  | Asp20Asn | Cardiomyopathy, hypertrophic | (Coppini et al., 2014; Walsh et al., 2017) |
|  | Arg21Leu | Cardiomyopathy, hypertrophic | (Alejandra Restrepo-Cordoba et al., 2017; Iglesias et al., 2021; Mendes de Almeida et al., 2017) |
|  | Ala22Thr | Cardiomyopathy, hypertrophic | (Otsuka et al., 2012; Walsh et al., 2017) |
|  | Glu23Asp | Cardiomyopathy, hypertrophic | (Ho et al., 2018) |
|  | Glu23Gln | Cardiomyopathy, dilated | (Hershberger et al., 2010) |
|  | Glu23Lys | Cardiomyopathy, hypertrophic | (Hayashi et al., 2018; Mazzarotto et al., 2020) |
|  | Ala25Thr | Cardiomyopathy, hypertrophic | (Ho et al., 2018) |
|  | Glu26Gln | Noncompaction, left | (Miszalski-Jamka et al., 2017) |
|  | Asp28Gln | Cardiomyopathy, hypertrophic | (Walsh et al., 2017) |
|  | Asp28Asn | Cardiomyopathy, hypertrophic | (Bos et al., 2014; Robyns et al., 2020; Walsh et al., 2017) |
|  | Asp28His | Cardiomyopathy, hypertrophic | (Hathaway et al., 2021; Walsh et al., 2017; Yoneda et al., 2021) |
|  | Ala31Thr | Cardiomyopathy, dilated | (Walsh et al., 2017) (Walsh et al., 2017) |
|  | Glu33Lys | Cardiomyopathy, dilated | (Deacon et al., 2019; Pugh et al., 2014; Walsh et al., 2017) |
|  | Arg35Ser | Cardiomyopathy, hypertrophic | (Pua et al., 2020) |
|  | Lys37Glu | Cardiomyopathy, non-compaction, left ventricular | (Chang et al., 2011; Takasaki et al., 2018) |
| *2b*  *(not in SMC)* | *Glu40Gln* | *Cardiomyopathy, dilated* | *(Khan et al., 2022)* |
|  | *Glu40Lys* | *Cardiomyopathy, dilated* | *(Borovikov et al., 2009; Mirza et al., 2005; Olson et al., 2001)* |
|  | *Glu40Term* | *Cardiomyopathy, dilated* | *(Walsh et al., 2017)* |
|  | *Glu54Lys* | *Cardiomyopathy, dilated* | *(Mirza et al., 2005; Olson et al., 2001; Rajan et al., 2007)* |
|  | *Asp55Asn* | *Cardiomyopathy, dilated* | *(Pugh et al., 2014)* |
|  | *Asp58Asn* | *Cardiomyopathy, hypertrophic* | *(Marschall et al., 2019)* |
|  | *Asp58His* | *Cardiomyopathy, hypertrophic* | *(Frisso et al., 2009; Orzechowski et al., 2014; Xiong et al., 2015)* |
|  | *Ser61Cys* | *Cardiomyopathy, hypertrophic* | *(Bos et al., 2014)* |
|  | *Glu62Gln* | *Cardiomyopathy, hypertrophic* | *(Gupte et al., 2015; Jongbloed et al., 2003; Orzechowski et al., 2014)* |
|  | *Ala63Val* | *Cardiomyopathy, hypertrophic* | *(Davis and Metzger, 2010; Heller et al., 2003; Yamauchi-Takihara et al., 1996)* |
|  | *Lys70Thr* | *Cardiomyopathy, hypertrophic* | *(Davis and Metzger, 2010; Heller et al., 2003; Yamauchi-Takihara et al., 1996)* |
|  | *Leu71Val* | *Cardiomyopathy, hypertrophic* | *(Alejandra Restrepo-Cordoba et al., 2017)* |
|  | *Ala74Ser* | *Sudden unexplained death* | *(Lin et al., 2017)* |
| 3  (Shared) | Asp80Asn | Cardiomyopathy, dilated | (Carnevale et al., 2020) |
|  | Asp80Gly | Cardiomyopathy, dilated | (Marschall et al., 2019) |
|  | Asp84Asn | Cardiomyopathy, dilated non-compaction | (Gupte et al., 2015; Orzechowski et al., 2014; van de Meerakker et al., 2013) |
|  | Asp84Glu | Cardiomyopathy, hypertrophic | (Walsh et al., 2017) |
|  | Asp84Tyr | Cardiomyopathy, hypertrophic | (Seo et al., 2020) |
|  | Val85Ile | Cardiomyopathy, hypertrophic | (Viswanathan et al., 2017) |
|  | Ala86Thr | Cardiomyopathy, dilated | (Chanavat et al., 2016) |
|  | Ala86Val | Cardiomyopathy, non-compaction, left ventricular | (Kuhnisch et al., 2019; Schultze-Berndt et al., 2021) |
|  | Ile92Thr | Cardiomyopathy, dilated | Hershberger (2010) Circ Cardiovasc Genet **3,** 155 (Walsh et al., 2017)[Additional report]Ware (2021) *J Am Heart Assoc* **10:** e017731 [Additional report] |
|  | Val95Ala | Cardiomyopathy, hypertrophic | Karibe (2001) Circulation **103,** 65Bai (2011) *Biophys J* **100:** 1014 [Functional characterisation]Mathur (2011) *Biochem Biophys Res Commun* **406:** 74 [Functional characterisation]3 more reference(s)... |
|  | Arg101Pro | Cardiomyopathy, hypertrophic | (Walsh et al., 2017) |
|  | Ala102Asp | Cardiomyopathy, hypertrophic | (Chida et al., 2017) |
|  | Thr108Ala | Cardiomyopathy, dilated | (Mehaney et al., 2022) |
|  | Gln111His | Cardiomyopathy, dilated | (Ji et al., 2015) |
|  | Leu113Val | Cardiomyopathy, dilated | (Cuenca et al., 2016; Pugh et al., 2014; Walsh et al., 2017) |
|  | Glu114Gln | Cardiomyopathy, dilated | (Man et al., 2022) |
|  | Glu114Gly | Cardiomyopathy, dilated | (Pugh et al., 2014; Walsh et al., 2017) |
|  | Glu115Lys | Cardiomyopathy, hypertrophic | (Alfares et al., 2015; Marschall et al., 2019; Walsh et al., 2017) |
|  | Ala120Val | Cardiomyopathy, dilated | (Augusto et al., 2020; Walsh et al., 2017) |
|  | Ser123Asn | Cardiomyopathy, hypertrophic | (Ho et al., 2018) |
|  | Ser123Thr | Cardiomyopathy, dilated | (Walsh et al., 2017) |
| 4  (Shared) | Gly126Ser | Cardiomyopathy, hypertrophic | (Chung et al., 2021; Kim et al., 2020) |
|  | Met127Leu | Cardiomyopathy, hypertrophic | (Pua et al., 2020) |
|  | Met127Lys | Cardiomyopathy, hypertrophic | (Zou et al., 2013) |
|  | Ile130Val | Pulmonary atresia | (England et al., 2017) |
|  | Glu138Lys | Cardiomyopathy | (Fokstuen et al., 2016) |
|  | Glu139Val | Cardiomyopathy, dilated | (Burstein et al., 2021; Pugh et al., 2014; Walsh et al., 2017) |
|  | Met141Ile | Cardiomyopathy, dilated | (Pugh et al., 2014; Quiat et al., 2020; Walsh et al., 2017) |
|  | Met141Leu | Cardiomyopathy, dilated | (Hey et al., 2020) |
|  | Met141Lys | Cardiomyopathy, hypertrophic | (Tran Vu et al., 2019) |
|  | Ile143Ser | Cardiomyopathy, hypertrophic | (Ho et al., 2018) |
|  | Glu145Gly | Cardiomyopathy, dilated | (Marinakis et al., 2021) |
|  | His153Asp | Cardiomyopathy, hypertrophic | (Alfares et al., 2015; Pugh et al., 2014; Walsh et al., 2017) |
|  | Ile154Thr | Cardiomyopathy, hypertrophic | (Walsh et al., 2017) |
|  | Asp159Asn | Ebstein anomaly, left ventricular noncompaction & heart failure, early onset | (Kelle et al., 2016; Marschall et al., 2019; Walsh et al., 2017) |
|  | Arg160His | Cardiomyopathy, non-compaction, left ventricular | (Guo et al., 2021; Hoedemaekers et al., 2010; Orzechowski et al., 2014) |
|  | Lys161Glu | Cardiomyopathy, hypertrophic | (Pugh et al., 2014; Walsh et al., 2017) |
|  | Glu164Gly | Cardiomyopathy, dilated | (Horvat et al., 2019) |
| 5  (Shared) | Ala166Thr | Cardiomyopathy, dilated, modifier of | (Burstein et al., 2021) |
|  | Ala166Val | Cardiomyopathy, dilated | (Hu et al., 2018) |
|  | Ile172Thr | Cardiomyopathy, hypertrophic | (Amendola et al., 2015; Andreasen et al., 2013; Van Driest et al., 2003) |
|  | Asp175Asn | Cardiomyopathy, hypertrophic | (Bing et al., 1997; Thierfelder et al., 1994; Yamauchi-Takihara et al., 1996) |
|  | Asp175Gly | Cardiomyopathy, hypertrophic | (Hathaway et al., 2021; Jaaskelainen et al., 2014) |
|  | Arg178His | Noncompaction, left ventricular | (Clark et al., 2019; Farnaes et al., 2018; Takasaki et al., 2018) |
|  | Glu180Gly | Cardiomyopathy, hypertrophic | (Bing et al., 1997; Thierfelder et al., 1994)] |
|  | Glu180Val | Cardiomyopathy, hypertrophic | (Matyushenko et al., 2017; Regitz-Zagrosek et al., 2000) |
|  | Ala183Val | Cardiomyopathy, | (Lopes et al., 2015) |
|  | Leu185Arg | Cardiomyopathy, hypertrophic | (Earing et al., 2003; Orzechowski et al., 2014; Van Driest et al., 2003) |
|  | Leu185Phe | Cardiomyopathy, dilated | (Hey et al., 2020) |
|  | Glu187Gln | Cardiomyopathy, hypertrophic | (Alfares et al., 2015; Walsh et al., 2017) |
| 6b  (Shared with TPM1.4) | Cys190Arg | Cardiomyopathy, hypertrophic | (Hathaway et al., 2021) |
|  | Glu192Lys | Cardiomyopathy, hypertrophic | (Orzechowski et al., 2014; Probst et al., 2011; Van Driest et al., 2003) |
|  | Glu195Lys | Kartagener syndrome | (Chen et al., 2020) |
|  | Val200Leu | Cardiomyopathy, dilated | (Nguyen et al., 2021) |
|  | Thr201Met | Cardiomyopathy, dilated | (Dorsch et al., 2021; Mazzarotto et al., 2020; van Spaendonck-Zwarts et al., 2013) |
|  | Asn202Ser | Cardiomyopathy, hypertrophic | (Norrish et al., 2019) |
|  | Lys205Arg | Cardiomyopathy, dilated | (Franaszczyk et al., 2020) |
|  | Lys205Gln | Cardiomyopathy, dilated, modifier of | (Burstein et al., 2021) |
|  | Gln210Arg | Cardiomyopathy, hypertrophic | (Filatova et al., 2021; Zou et al., 2013) |
|  | Ala211Gly | Cardiomyopathy, dilated | (Lakdawala et al., 2012; Walsh et al., 2017) |
|  | Glu212Val | Cardiomyopathy, hypertrophic | (Burns et al., 2017) |
| 7  (Shared) | Ser215Leu | Cardiomyopathy, hypertrophic | (Gupte et al., 2015; Morita et al., 2008; Viswanathan et al., 2017) |
|  | Gln216Arg | Left ventricular non-compaction | (Ross et al., 2020) |
|  | Asp219Asn | Cardiomyopathy, hypertrophic | (Coppini et al., 2014; Walsh et al., 2017) |
|  | Tyr221Cys | Cardiomyopathy, hypertrophic | (Nakashima et al., 2020) |
|  | Lys226Arg | Cardiomyopathy, hypertrophic | (van Lint et al., 2019; Walsh et al., 2017) |
|  | Lys226Gln | Cardiomyopathy, hypertrophic | (Walsh et al., 2017) |
|  | Ser229Phe | Atrial septal defect | (England et al., 2017) |
|  | Asp230Asn | Cardiomyopathy, dilated | (Jordan et al., 2011; Lakdawala et al., 2010; Memo et al., 2013) |
|  | Leu232Arg | Cardiomyopathy, dilated | (Walsh et al., 2017) |
|  | Lys233Asn | Cardiomyopathy, hypertrophic | (Walsh et al., 2017) |
| 8  (Shared) | Arg238Gln | Noncompaction, left ventricular | (Hirono et al., 2020; Wang et al., 2017) |
|  | Arg238Trp | Cardiomyopathy, dilated | (Walsh et al., 2017; Zimmerman et al., 2010) |
|  | Ala239Thr | Cardiomyopathy, dilated | (Hershberger et al., 2010; Walsh et al., 2017) |
|  | Ala242Val | Cardiomyopathy, non-compaction, left ventricular | (Hershberger et al., 2010; Pugh et al., 2014; Tian et al., 2015; Walsh et al., 2017) |
|  | Ser245Leu | Cardiomyopathy, dilated | (Walsh et al., 2017) |
|  | Lys248Glu | Cardiomyopathy, non-compaction, left ventricular | (Orzechowski et al., 2014; Probst et al., 2011) |
|  | Leu249Trp | Cardiomyopathy, hypertrophic | (Lopes et al., 2015; Walsh et al., 2017) |
|  | Ser252Asn | Cardiomyopathy, hypertrophic | (Marschall et al., 2019) |
|  | Ser252Thr | Cardiomyopathy, hypertrophic | (Norrish et al., 2019) |
|  | Ile253Thr | Cardiomyopathy, dilated | (Sousa et al., 2019) |
|  | Asp254Glu | Cardiomyopathy, hypertrophic | (Alfares et al., 2015) |
|  | Asp254Gly | Cardiomyopathy, hypertrophic | (Lopes et al., 2015; Marschall et al., 2019; Walsh et al., 2017) |
| *9a*  *(not shared)* | *Asp258Glu* | *Cardiomyopathy, hypertrophic* | *(Walsh et al., 2017)* |
|  | *Gln263Glu* | *Sudden cardiac death* | *(Chanavat et al., 2016; Robyns et al., 2020)* |
|  | *Lys264Arg* | *Cardiomyopathy, hypertrophic* | *(Kim et al., 2020; Lopes et al., 2015)* |
|  | *Lys264Glu* | *Cardiomyopathy, hypertrophic* | *(Alfares et al., 2015; Walsh et al., 2017)* |
|  | *Lys266Arg* | *Cardiomyopathy, hypertrophic* | *(Walsh et al., 2017)Burstein (2021) Pediatr Res* ***89:*** *1470* |
|  | *Lys268Arg* | *Cardiomyopathy, arrhythmogenic* | *(Poloni et al., 2019)* |
|  | *Glu272Gly* | *Noncompaction, left ventricular* | *(Miller et al., 2017; Parrott et al., 2020)* |
|  | *Asp275His* | *Cardiomyopathy, non-compaction, left ventricular* | *(Bainbridge et al., 2015)* |
|  | *Ala277Thr* | *Cardiomyopathy, dilated* | *(Verdonschot et al., 2020)* |
|  | *Ala277Val* | *Cardiomyopathy, dilated* | *(Hershberger et al., 2010)* |
|  | *Leu278Phe* | *Cardiomyopathy, hypertrophic* | *(Ho et al., 2018)* |
|  | *Asn279His* | *Cardiomyopathy, restrictive* | *(Caleshu et al., 2011; Walsh et al., 2017)* |
|  | *Met281Thr* | *Cardiomyopathy, hypertrophic* | *(Gupte et al., 2015; Van Driest et al., 2003)* |
|  | *Met281Val* | *Cardiomyopathy, hypertrophic* | *(Marston et al., 2013; Mendes de Almeida et al., 2017; Walsh et al., 2017)* |
|  | *Thr282Ser* | *Cardiomyopathy, dilated* | *(Wilson et al., 2015)* |
|  | *Ile284Thr* | *Cardiomyopathy, hypertrophic* | *(Robyns et al., 2020)****,*** |
|  | *Ile284Val* | *Cardiomyopathy, hypertrophic* | *(Helms et al., 2014; Witjas-Paalberends et al., 2013)* |

**References**

Alejandra Restrepo-Cordoba, M., O. Campuzano, T. Ripoll-Vera, M. Cobo-Marcos, I. Mademont-Soler, J.M. Gamez, F. Dominguez, E. Gonzalez-Lopez, L. Padron-Barthe, E. Lara-Pezzi, L. Alonso-Pulpon, R. Brugada, and P. Garcia-Pavia. 2017. Usefulness of Genetic Testing in Hypertrophic Cardiomyopathy: an Analysis Using Real-World Data. *J Cardiovasc Transl Res*. 10:35-46.

Alfares, A.A., M.A. Kelly, G. McDermott, B.H. Funke, M.S. Lebo, S.B. Baxter, J. Shen, H.M. McLaughlin, E.H. Clark, L.J. Babb, S.W. Cox, S.R. DePalma, C.Y. Ho, J.G. Seidman, C.E. Seidman, and H.L. Rehm. 2015. Results of clinical genetic testing of 2,912 probands with hypertrophic cardiomyopathy: expanded panels offer limited additional sensitivity. *Genet Med*. 17:880-888.

Amendola, L.M., M.O. Dorschner, P.D. Robertson, J.S. Salama, R. Hart, B.H. Shirts, M.L. Murray, M.J. Tokita, C.J. Gallego, D.S. Kim, J.T. Bennett, D.R. Crosslin, J. Ranchalis, K.L. Jones, E.A. Rosenthal, E.R. Jarvik, A. Itsara, E.H. Turner, D.S. Herman, J. Schleit, A. Burt, S.M. Jamal, J.L. Abrudan, A.D. Johnson, L.K. Conlin, M.C. Dulik, A. Santani, D.R. Metterville, M. Kelly, A.K. Foreman, K. Lee, K.D. Taylor, X. Guo, K. Crooks, L.A. Kiedrowski, L.J. Raffel, O. Gordon, K. Machini, R.J. Desnick, L.G. Biesecker, S.A. Lubitz, S. Mulchandani, G.M. Cooper, S. Joffe, C.S. Richards, Y. Yang, J.I. Rotter, S.S. Rich, C.J. O'Donnell, J.S. Berg, N.B. Spinner, J.P. Evans, S.M. Fullerton, K.A. Leppig, R.L. Bennett, T. Bird, V.P. Sybert, W.M. Grady, H.K. Tabor, J.H. Kim, M.J. Bamshad, B. Wilfond, A.G. Motulsky, C.R. Scott, C.C. Pritchard, T.D. Walsh, W. Burke, W.H. Raskind, P. Byers, F.M. Hisama, H. Rehm, D.A. Nickerson, and G.P. Jarvik. 2015. Actionable exomic incidental findings in 6503 participants: challenges of variant classification. *Genome Res*. 25:305-315.

Andreasen, C., J.B. Nielsen, L. Refsgaard, A.G. Holst, A.H. Christensen, L. Andreasen, A. Sajadieh, S. Haunso, J.H. Svendsen, and M.S. Olesen. 2013. New population-based exome data are questioning the pathogenicity of previously cardiomyopathy-associated genetic variants. *Eur J Hum Genet*. 21:918-928.

Augusto, J.B., R. Eiros, E. Nakou, S. Moura-Ferreira, T.A. Treibel, G. Captur, M.M. Akhtar, A. Protonotarios, T.D. Gossios, K. Savvatis, P. Syrris, S. Mohiddin, J.C. Moon, P.M. Elliott, and L.R. Lopes. 2020. Dilated cardiomyopathy and arrhythmogenic left ventricular cardiomyopathy: a comprehensive genotype-imaging phenotype study. *Eur Heart J Cardiovasc Imaging*. 21:326-336.

Bainbridge, M.N., E.E. Davis, W.Y. Choi, A. Dickson, H.R. Martinez, M. Wang, H. Dinh, D.M. Muzny, R. Pignatelli, N. Katsanis, E. Boerwinkle, R.A. Gibbs, and J.L. Jefferies. 2015. Loss of Function Mutations in NNT Are Associated With Left Ventricular Noncompaction. *Circ Cardiovasc Genet*. 8:544-552.

Bing, W., C.S. Redwood, I.F. Purcell, G. Esposito, H. Watkins, and S.B. Marston. 1997. Effects of two hypertrophic cardiomyopathy mutations in alpha-tropomyosin, Asp175Asn and Glu180Gly, on Ca2+ regulation of thin filament motility. *Biochem Biophys Res Commun*. 236:760-764.

Borovikov, Y.S., O.E. Karpicheva, G.A. Chudakova, P. Robinson, and C.S. Redwood. 2009. Dilated cardiomyopathy mutations in alpha-tropomyosin inhibit its movement during the ATPase cycle. *Biochem Biophys Res Commun*. 381:403-406.

Bos, J.M., M.L. Will, B.J. Gersh, T.M. Kruisselbrink, S.R. Ommen, and M.J. Ackerman. 2014. Characterization of a phenotype-based genetic test prediction score for unrelated patients with hypertrophic cardiomyopathy. *Mayo Clin Proc*. 89:727-737.

Burns, C., R.D. Bagnall, L. Lam, C. Semsarian, and J. Ingles. 2017. Multiple Gene Variants in Hypertrophic Cardiomyopathy in the Era of Next-Generation Sequencing. *Circ Cardiovasc Genet*. 10.

Burstein, D.S., J.W. Gaynor, H. Griffis, A. Ritter, M.J.O. Connor, J.W. Rossano, K.Y. Lin, and R.C. Ahrens-Nicklas. 2021. Genetic variant burden and adverse outcomes in pediatric cardiomyopathy. *Pediatr Res*. 89:1470-1476.

Caleshu, C., R. Sakhuja, R.L. Nussbaum, N.B. Schiller, P.C. Ursell, C. Eng, T. De Marco, D. McGlothlin, E.G. Burchard, and J.E. Rame. 2011. Furthering the link between the sarcomere and primary cardiomyopathies: restrictive cardiomyopathy associated with multiple mutations in genes previously associated with hypertrophic or dilated cardiomyopathy. *Am J Med Genet A*. 155A:2229-2235.

Carnevale, A., S. Rosas-Madrigal, R. Rosendo-Gutierrez, E. Lopez-Mora, S. Romero-Hidalgo, N. Avila-Vazzini, L. Jacobo-Albavera, M. Dominguez-Perez, G. Vargas-Alarcon, F. Perez-Villatoro, J.I. Navarrete-Martinez, and M.T. Villarreal-Molina. 2020. Genomic study of dilated cardiomyopathy in a group of Mexican patients using site-directed next generation sequencing. *Mol Genet Genomic Med*. 8:e1504.

Chanavat, V., A. Janin, and G. Millat. 2016. A fast and cost-effective molecular diagnostic tool for genetic diseases involved in sudden cardiac death. *Clin Chim Acta*. 453:80-85.

Chang, B., T. Nishizawa, M. Furutani, A. Fujiki, M. Tani, M. Kawaguchi, K. Ibuki, K. Hirono, H. Taneichi, K. Uese, Y. Onuma, N.E. Bowles, F. Ichida, H. Inoue, R. Matsuoka, T. Miyawaki, and c. Noncompaction study. 2011. Identification of a novel TPM1 mutation in a family with left ventricular noncompaction and sudden death. *Mol Genet Metab*. 102:200-206.

Chen, Y.L., Y.X. Zhang, X.F. Yang, J. Chen, X.T. Li, M.H. Huang, J.W. Ruan, and Q. Lin. 2020. [Application value of whole exome sequencing in critically ill neonates with inherited diseases]. *Zhongguo Dang Dai Er Ke Za Zhi*. 22:1261-1266.

Chida, A., K. Inai, H. Sato, E. Shimada, T. Nishizawa, M. Shimada, M. Furutani, Y. Furutani, Y. Kawamura, M. Sugimoto, J. Ishihara, M. Fujiwara, T. Soga, M. Kawana, S. Fuji, S. Tateno, K. Kuraishi, S. Kogaki, M. Nishimura, M. Ayusawa, F. Ichida, H. Yamazawa, R. Matsuoka, S. Nonoyama, and T. Nakanishi. 2017. Prognostic predictive value of gene mutations in Japanese patients with hypertrophic cardiomyopathy. *Heart Vessels*. 32:700-707.

Chung, H., Y. Kim, C.H. Park, J.Y. Kim, P.K. Min, Y.W. Yoon, T.H. Kim, B.K. Lee, B.K. Hong, S.J. Rim, H.M. Kwon, K.A. Lee, and E.Y. Choi. 2021. Effect of sarcomere and mitochondria-related mutations on myocardial fibrosis in patients with hypertrophic cardiomyopathy. *J Cardiovasc Magn Reson*. 23:18.

Clark, M.M., A. Hildreth, S. Batalov, Y. Ding, S. Chowdhury, K. Watkins, K. Ellsworth, B. Camp, C.I. Kint, C. Yacoubian, L. Farnaes, M.N. Bainbridge, C. Beebe, J.J.A. Braun, M. Bray, J. Carroll, J.A. Cakici, S.A. Caylor, C. Clarke, M.P. Creed, J. Friedman, A. Frith, R. Gain, M. Gaughran, S. George, S. Gilmer, J. Gleeson, J. Gore, H. Grunenwald, R.L. Hovey, M.L. Janes, K. Lin, P.D. McDonagh, K. McBride, P. Mulrooney, S. Nahas, D. Oh, A. Oriol, L. Puckett, Z. Rady, M.G. Reese, J. Ryu, L. Salz, E. Sanford, L. Stewart, N. Sweeney, M. Tokita, L. Van Der Kraan, S. White, K. Wigby, B. Williams, T. Wong, M.S. Wright, C. Yamada, P. Schols, J. Reynders, K. Hall, D. Dimmock, N. Veeraraghavan, T. Defay, and S.F. Kingsmore. 2019. Diagnosis of genetic diseases in seriously ill children by rapid whole-genome sequencing and automated phenotyping and interpretation. *Sci Transl Med*. 11.

Colpan, M., T. Ly, S. Grover, D. Tolkatchev, and A.S. Kostyukova. 2017. The cardiomyopathy-associated K15N mutation in tropomyosin alters actin filament pointed end dynamics. *Arch Biochem Biophys*. 630:18-26.

Coppini, R., C.Y. Ho, E. Ashley, S. Day, C. Ferrantini, F. Girolami, B. Tomberli, S. Bardi, F. Torricelli, F. Cecchi, A. Mugelli, C. Poggesi, J. Tardiff, and I. Olivotto. 2014. Clinical phenotype and outcome of hypertrophic cardiomyopathy associated with thin-filament gene mutations. *J Am Coll Cardiol*. 64:2589-2600.

Cuenca, S., M.J. Ruiz-Cano, J.R. Gimeno-Blanes, A. Jurado, C. Salas, I. Gomez-Diaz, L. Padron-Barthe, J.J. Grillo, C. Vilches, J. Segovia, D. Pascual-Figal, E. Lara-Pezzi, L. Monserrat, L. Alonso-Pulpon, P. Garcia-Pavia, and N. Inherited Cardiac Diseases Program of the Spanish Cardiovascular Research. 2016. Genetic basis of familial dilated cardiomyopathy patients undergoing heart transplantation. *J Heart Lung Transplant*. 35:625-635.

Davis, J., and J.M. Metzger. 2010. Combinatorial effects of double cardiomyopathy mutant alleles in rodent myocytes: a predictive cellular model of myofilament dysregulation in disease. *PLoS One*. 5:e9140.

Deacon, D.C., C.L. Happe, C. Chen, N. Tedeschi, A.M. Manso, T. Li, N.D. Dalton, Q. Peng, E.N. Farah, Y. Gu, K.P. Tenerelli, V.D. Tran, J. Chen, K.L. Peterson, N.J. Schork, E.D. Adler, A.J. Engler, R.S. Ross, and N.C. Chi. 2019. Combinatorial interactions of genetic variants in human cardiomyopathy. *Nat Biomed Eng*. 3:147-157.

Dorsch, L.M., D.W.D. Kuster, J.D.H. Jongbloed, L.G. Boven, K.Y. van Spaendonck-Zwarts, A.J.H. Suurmeijer, A. Vink, G.J. du Marchie Sarvaas, M.P. van den Berg, J. van der Velden, B. Brundel, and P.A. van der Zwaag. 2021. The effect of tropomyosin variants on cardiomyocyte function and structure that underlie different clinical cardiomyopathy phenotypes. *Int J Cardiol*. 323:251-258.

Earing, M.G., M.J. Ackerman, and P.W. O'Leary. 2003. Diastolic ventricular dysfunction as a marker for hypertrophic cardiomyopathy in a family with a novel alpha-tropomyosin mutation. *J Am Soc Echocardiogr*. 16:698-702.

England, J., J. Granados-Riveron, L. Polo-Parada, D. Kuriakose, C. Moore, J.D. Brook, C.S. Rutland, K. Setchfield, C. Gell, T.K. Ghosh, F. Bu'Lock, C. Thornborough, E. Ehler, and S. Loughna. 2017. Tropomyosin 1: Multiple roles in the developing heart and in the formation of congenital heart defects. *J Mol Cell Cardiol*. 106:1-13.

Farnaes, L., A. Hildreth, N.M. Sweeney, M.M. Clark, S. Chowdhury, S. Nahas, J.A. Cakici, W. Benson, R.H. Kaplan, R. Kronick, M.N. Bainbridge, J. Friedman, J.J. Gold, Y. Ding, N. Veeraraghavan, D. Dimmock, and S.F. Kingsmore. 2018. Rapid whole-genome sequencing decreases infant morbidity and cost of hospitalization. *NPJ Genom Med*. 3:10.

Filatova, E.V., N.S. Krylova, I.N. Vlasov, M.S. Maslova, N.G. Poteshkina, P.A. Slominsky, and M.I. Shadrina. 2021. Targeted exome analysis of Russian patients with hypertrophic cardiomyopathy. *Mol Genet Genomic Med*. 9:e1808.

Fokstuen, S., P. Makrythanasis, E. Hammar, M. Guipponi, E. Ranza, K. Varvagiannis, F.A. Santoni, M. Albarca-Aguilera, M.E. Poleggi, F. Couchepin, C. Brockmann, A. Mauron, S.A. Hurst, C. Moret, C. Gehrig, A. Vannier, J. Bevillard, T. Araud, S. Gimelli, E. Stathaki, A. Paoloni-Giacobino, A. Bottani, F. Sloan-Bena, L.D. Sizonenko, M. Mostafavi, H. Hamamy, T. Nouspikel, J.L. Blouin, and S.E. Antonarakis. 2016. Experience of a multidisciplinary task force with exome sequencing for Mendelian disorders. *Hum Genomics*. 10:24.

Franaszczyk, M., G. Truszkowska, P. Chmielewski, M. Rydzanicz, J. Kosinska, T. Rywik, A. Biernacka, M. Spiewak, G. Kostrzewa, M. Stepien-Wojno, P. Stawinski, M. Bilinska, P. Krajewski, T. Zielinski, A. Lutynska, Z.T. Bilinska, and R. Ploski. 2020. Analysis of De Novo Mutations in Sporadic Cardiomyopathies Emphasizes Their Clinical Relevance and Points to Novel Candidate Genes. *J Clin Med*. 9.

Frisso, G., G. Limongelli, G. Pacileo, A. Del Giudice, L. Forgione, P. Calabro, M. Iacomino, N. Detta, L.M. Di Fonzo, V. Maddaloni, R. Calabro, and F. Salvatore. 2009. A child cohort study from southern Italy enlarges the genetic spectrum of hypertrophic cardiomyopathy. *Clin Genet*. 76:91-101.

Gomez, J., J.R. Reguero, C. Moris, M. Martin, V. Alvarez, B. Alonso, S. Iglesias, and E. Coto. 2014. Mutation analysis of the main hypertrophic cardiomyopathy genes using multiplex amplification and semiconductor next-generation sequencing. *Circ J*. 78:2963-2971.

Guo, L., S. Torii, R. Fernandez, R.E. Braumann, D.T. Fuller, K.H. Paek, N.V. Gadhoke, K.A. Maloney, K. Harris, C.M. Mayhew, R. Zarpak, L.M. Stevens, B.J. Gaynor, H. Jinnouchi, A. Sakamoto, Y. Sato, H. Mori, M.D. Kutyna, P.J. Lee, L.M. Weinstein, C.J. Collado-Rivera, B.B. Ali, D.R. Atmakuri, R. Dhingra, E.L.B. Finn, M.W. Bell, M. Lynch, A. Cornelissen, S.H. Kuntz, J.H. Park, R. Kutys, J.E. Park, L. Wang, S.N. Hong, A. Gupta, J.L. Hall, F.D. Kolodgie, M.E. Romero, L.J.B. Jeng, B.D. Mitchell, D. Surve, D.R. Fowler, C.C. Hong, R. Virmani, and A.V. Finn. 2021. Genetic Variants Associated With Unexplained Sudden Cardiac Death in Adult White and African American Individuals. *JAMA Cardiol*. 6:1013-1022.

Gupte, T.M., F. Haque, B. Gangadharan, M.S. Sunitha, S. Mukherjee, S. Anandhan, D.S. Rani, N. Mukundan, A. Jambekar, K. Thangaraj, R. Sowdhamini, R.F. Sommese, S. Nag, J.A. Spudich, and J.A. Mercer. 2015. Mechanistic heterogeneity in contractile properties of alpha-tropomyosin (TPM1) mutants associated with inherited cardiomyopathies. *J Biol Chem*. 290:7003-7015.

Hathaway, J., K. Helio, I. Saarinen, J. Tallila, E.H. Seppala, S. Tuupanen, H. Turpeinen, T. Kangas-Kontio, J. Schleit, J. Tommiska, V. Kytola, M. Valori, M. Muona, J. Sistonen, M. Gentile, P. Salmenpera, S. Myllykangas, J. Paananen, T.P. Alastalo, T. Helio, and J. Koskenvuo. 2021. Diagnostic yield of genetic testing in a heterogeneous cohort of 1376 HCM patients. *BMC Cardiovasc Disord*. 21:126.

Hayashi, T., K. Tanimoto, K. Hirayama-Yamada, E. Tsuda, M. Ayusawa, S. Nunoda, A. Hosaki, and A. Kimura. 2018. Genetic background of Japanese patients with pediatric hypertrophic and restrictive cardiomyopathy. *J Hum Genet*. 63:989-996.

Heller, M.J., M. Nili, E. Homsher, and L.S. Tobacman. 2003. Cardiomyopathic tropomyosin mutations that increase thin filament Ca2+ sensitivity and tropomyosin N-domain flexibility. *J Biol Chem*. 278:41742-41748.

Helms, A.S., F.M. Davis, D. Coleman, S.N. Bartolone, A.A. Glazier, F. Pagani, J.M. Yob, S. Sadayappan, E. Pedersen, R. Lyons, M.V. Westfall, R. Jones, M.W. Russell, and S.M. Day. 2014. Sarcomere mutation-specific expression patterns in human hypertrophic cardiomyopathy. *Circ Cardiovasc Genet*. 7:434-443.

Hershberger, R.E., N. Norton, A. Morales, D. Li, J.D. Siegfried, and J. Gonzalez-Quintana. 2010. Coding sequence rare variants identified in MYBPC3, MYH6, TPM1, TNNC1, and TNNI3 from 312 patients with familial or idiopathic dilated cardiomyopathy. *Circ Cardiovasc Genet*. 3:155-161.

Hey, T.M., T.B. Rasmussen, T. Madsen, M.M. Aagaard, M. Harbo, H. Molgaard, S.K. Nielsen, J. Haas, B. Meder, J.E. Moller, H. Eiskjaer, and J. Mogensen. 2020. Clinical and Genetic Investigations of 109 Index Patients With Dilated Cardiomyopathy and 445 of Their Relatives. *Circ Heart Fail*. 13:e006701.

Hirono, K., Y. Hata, N. Miyao, M. Okabe, S. Takarada, H. Nakaoka, K. Ibuki, S. Ozawa, H. Origasa, N. Nishida, F. Ichida, and L.s. collaborates*. 2020. Increased Burden of Ion Channel Gene Variants Is Related to Distinct Phenotypes in Pediatric Patients With Left Ventricular Noncompaction. *Circ Genom Precis Med*. 13:e002940.

Ho, C.Y., S.M. Day, E.A. Ashley, M. Michels, A.C. Pereira, D. Jacoby, A.L. Cirino, J.C. Fox, N.K. Lakdawala, J.S. Ware, C.A. Caleshu, A.S. Helms, S.D. Colan, F. Girolami, F. Cecchi, C.E. Seidman, G. Sajeev, J. Signorovitch, E.M. Green, and I. Olivotto. 2018. Genotype and Lifetime Burden of Disease in Hypertrophic Cardiomyopathy: Insights from the Sarcomeric Human Cardiomyopathy Registry (SHaRe). *Circulation*. 138:1387-1398.

Hoedemaekers, Y.M., K. Caliskan, M. Michels, I. Frohn-Mulder, J.J. van der Smagt, J.E. Phefferkorn, M.W. Wessels, F.J. ten Cate, E.J. Sijbrands, D. Dooijes, and D.F. Majoor-Krakauer. 2010. The importance of genetic counseling, DNA diagnostics, and cardiologic family screening in left ventricular noncompaction cardiomyopathy. *Circ Cardiovasc Genet*. 3:232-239.

Horvat, C., R. Johnson, L. Lam, J. Munro, F. Mazzarotto, A.M. Roberts, D.S. Herman, M. Parfenov, A. Haghighi, B. McDonough, S.R. DePalma, A.M. Keogh, P.S. Macdonald, C.S. Hayward, A. Roberts, P.J.R. Barton, L.E. Felkin, E. Giannoulatou, S.A. Cook, J.G. Seidman, C.E. Seidman, and D. Fatkin. 2019. A gene-centric strategy for identifying disease-causing rare variants in dilated cardiomyopathy. *Genet Med*. 21:133-143.

Hu, X., N. Li, Y. Xu, G. Li, T. Yu, R.E. Yao, L. Fu, J. Wang, L. Yin, Y. Yin, Y. Wang, X. Jin, X. Wang, J. Wang, and Y. Shen. 2018. Proband-only medical exome sequencing as a cost-effective first-tier genetic diagnostic test for patients without prior molecular tests and clinical diagnosis in a developing country: the China experience. *Genet Med*. 20:1045-1053.

Iglesias, M., T. Ripoll-Vera, C. Perez-Luengo, A.B. Garcia, S. Moyano, J.C. Canos, J.C. Borondo, J. Alvarez, D. Heine-Suner, and B. Barcelo. 2021. Diagnostic Yield of Genetic Testing in Sudden Cardiac Death with Autopsy Findings of Uncertain Significance. *J Clin Med*. 10.

Jaaskelainen, P., T. Helio, K. Aalto-Setala, M. Kaartinen, E. Ilveskoski, L. Hamalainen, J. Melin, S. Karkkainen, K. Peuhkurinen, M.S. Nieminen, M. Laakso, H.C.M.S.G. Fin, and J. Kuusisto. 2014. A new common mutation in the cardiac beta-myosin heavy chain gene in Finnish patients with hypertrophic cardiomyopathy. *Ann Med*. 46:424-429.

Ji, Y., Y. Li, H. Zhang, X. Zhou, Y. Zhang, J. Li, Q. Xing, J. Zhang, Y. Hong, and B. Tang. 2015. [TPM1 gene mutation is associated with dilated cardiomyopathy in Kazaks in Xinjiang]. *Zhonghua Xin Xue Guan Bing Za Zhi*. 43:521-526.

Jongbloed, R.J., C.L. Marcelis, P.A. Doevendans, J.M. Schmeitz-Mulkens, W.G. Van Dockum, J.P. Geraedts, and H.J. Smeets. 2003. Variable clinical manifestation of a novel missense mutation in the alpha-tropomyosin (TPM1) gene in familial hypertrophic cardiomyopathy. *J Am Coll Cardiol*. 41:981-986.

Jordan, D.M., A. Kiezun, S.M. Baxter, V. Agarwala, R.C. Green, M.F. Murray, T. Pugh, M.S. Lebo, H.L. Rehm, B.H. Funke, and S.R. Sunyaev. 2011. Development and validation of a computational method for assessment of missense variants in hypertrophic cardiomyopathy. *Am J Hum Genet*. 88:183-192.

Kelle, A.M., S.J. Bentley, L.O. Rohena, A.K. Cabalka, and T.M. Olson. 2016. Ebstein anomaly, left ventricular non-compaction, and early onset heart failure associated with a de novo alpha-tropomyosin gene mutation. *Am J Med Genet A*. 170:2186-2190.

Khan, R.S., E. Pahl, L. Dellefave-Castillo, K. Rychlik, A. Ing, K.L. Yap, C. Brew, J.R. Johnston, E.M. McNally, and G. Webster. 2022. Genotype and Cardiac Outcomes in Pediatric Dilated Cardiomyopathy. *J Am Heart Assoc*. 11:e022854.

Kim, H.Y., J.E. Park, S.C. Lee, E.S. Jeon, Y.K. On, S.M. Kim, Y.H. Choe, C.S. Ki, J.W. Kim, and K.H. Kim. 2020. Genotype-Related Clinical Characteristics and Myocardial Fibrosis and their Association with Prognosis in Hypertrophic Cardiomyopathy. *J Clin Med*. 9.

Kuhnisch, J., C. Herbst, N. Al-Wakeel-Marquard, J. Dartsch, M. Holtgrewe, A. Baban, G. Mearini, J. Hardt, K. Kolokotronis, B. Gerull, L. Carrier, D. Beule, S. Schubert, D. Messroghli, F. Degener, F. Berger, and S. Klaassen. 2019. Targeted panel sequencing in pediatric primary cardiomyopathy supports a critical role of TNNI3. *Clin Genet*. 96:549-559.

Lakdawala, N.K., L. Dellefave, C.S. Redwood, E. Sparks, A.L. Cirino, S. Depalma, S.D. Colan, B. Funke, R.S. Zimmerman, P. Robinson, H. Watkins, C.E. Seidman, J.G. Seidman, E.M. McNally, and C.Y. Ho. 2010. Familial dilated cardiomyopathy caused by an alpha-tropomyosin mutation: the distinctive natural history of sarcomeric dilated cardiomyopathy. *J Am Coll Cardiol*. 55:320-329.

Lakdawala, N.K., B.H. Funke, S. Baxter, A.L. Cirino, A.E. Roberts, D.P. Judge, N. Johnson, N.J. Mendelsohn, C. Morel, M. Care, W.K. Chung, C. Jones, A. Psychogios, E. Duffy, H.L. Rehm, E. White, J.G. Seidman, C.E. Seidman, and C.Y. Ho. 2012. Genetic testing for dilated cardiomyopathy in clinical practice. *J Card Fail*. 18:296-303.

Lin, Y., N. Williams, D. Wang, W. Coetzee, B. Zhou, L.S. Eng, S.Y. Um, R. Bao, O. Devinsky, T.V. McDonald, B.A. Sampson, and Y. Tang. 2017. Applying High-Resolution Variant Classification to Cardiac Arrhythmogenic Gene Testing in a Demographically Diverse Cohort of Sudden Unexplained Deaths. *Circ Cardiovasc Genet*. 10.

Lopes, L.R., P. Syrris, O.P. Guttmann, C. O'Mahony, H.C. Tang, C. Dalageorgou, S. Jenkins, M. Hubank, L. Monserrat, W.J. McKenna, V. Plagnol, and P.M. Elliott. 2015. Novel genotype-phenotype associations demonstrated by high-throughput sequencing in patients with hypertrophic cardiomyopathy. *Heart*. 101:294-301.

Man, Y., C. Yi, M. Fan, T. Yang, P. Liu, S. Liu, and G. Wang. 2022. Identification of a novel missense mutation in the TPM1 gene via exome sequencing in a Chinese family with dilated cardiomyopathy: A case report and literature review. *Medicine (Baltimore)*. 101:e28551.

Marinakis, N.M., M. Svingou, D. Veltra, K. Kekou, C. Sofocleous, F.N. Tilemis, K. Kosma, E. Tsoutsou, H. Fryssira, and J. Traeger-Synodinos. 2021. Phenotype-driven variant filtration strategy in exome sequencing toward a high diagnostic yield and identification of 85 novel variants in 400 patients with rare Mendelian disorders. *Am J Med Genet A*. 185:2561-2571.

Marschall, C., A. Moscu-Gregor, and H.G. Klein. 2019. Variant panorama in 1,385 index patients and sensitivity of expanded next-generation sequencing panels in arrhythmogenic disorders. *Cardiovasc Diagn Ther*. 9:S292-S298.

Marston, S., M. Memo, A. Messer, M. Papadaki, K. Nowak, E. McNamara, R. Ong, M. El-Mezgueldi, X. Li, and W. Lehman. 2013. Mutations in repeating structural motifs of tropomyosin cause gain of function in skeletal muscle myopathy patients. *Hum Mol Genet*. 22:4978-4987.

Matyushenko, A.M., D.V. Shchepkin, G.V. Kopylova, K.E. Popruga, N.V. Artemova, A.V. Pivovarova, S.Y. Bershitsky, and D.I. Levitsky. 2017. Structural and Functional Effects of Cardiomyopathy-Causing Mutations in the Troponin T-Binding Region of Cardiac Tropomyosin. *Biochemistry*. 56:250-259.

Mazzarotto, F., U. Tayal, R.J. Buchan, W. Midwinter, A. Wilk, N. Whiffin, R. Govind, E. Mazaika, A. de Marvao, T.J.W. Dawes, L.E. Felkin, M. Ahmad, P.I. Theotokis, E. Edwards, A.Y. Ing, K.L. Thomson, L.L.H. Chan, D. Sim, A.J. Baksi, A. Pantazis, A.M. Roberts, H. Watkins, B. Funke, D.P. O'Regan, I. Olivotto, P.J.R. Barton, S.K. Prasad, S.A. Cook, J.S. Ware, and R. Walsh. 2020. Reevaluating the Genetic Contribution of Monogenic Dilated Cardiomyopathy. *Circulation*. 141:387-398.

Mehaney, D.A., A. Haghighi, A.K. Embaby, R.A. Zeyada, R.K. Darwish, N.S. Elfeel, M. Abouelhoda, S.A. El-Saiedi, N.A. Gohar, and Z.S. Seliem. 2022. Molecular analysis of dilated and left ventricular noncompaction cardiomyopathies in Egyptian children. *Cardiol Young*. 32:295-300.

Memo, M., M.C. Leung, D.G. Ward, C. dos Remedios, S. Morimoto, L. Zhang, G. Ravenscroft, E. McNamara, K.J. Nowak, S.B. Marston, and A.E. Messer. 2013. Familial dilated cardiomyopathy mutations uncouple troponin I phosphorylation from changes in myofibrillar Ca(2)(+) sensitivity. *Cardiovasc Res*. 99:65-73.

Mendes de Almeida, R., J. Tavares, S. Martins, T. Carvalho, F.J. Enguita, D. Brito, M. Carmo-Fonseca, and L.R. Lopes. 2017. Whole gene sequencing identifies deep-intronic variants with potential functional impact in patients with hypertrophic cardiomyopathy. *PLoS One*. 12:e0182946.

Miller, E.M., R.B. Hinton, R. Czosek, A. Lorts, A. Parrott, A.R. Shikany, R.F. Ittenbach, and S.M. Ware. 2017. Genetic Testing in Pediatric Left Ventricular Noncompaction. *Circ Cardiovasc Genet*. 10.

Mirza, M., S. Marston, R. Willott, C. Ashley, J. Mogensen, W. McKenna, P. Robinson, C. Redwood, and H. Watkins. 2005. Dilated cardiomyopathy mutations in three thin filament regulatory proteins result in a common functional phenotype. *J Biol Chem*. 280:28498-28506.

Miszalski-Jamka, K., J.L. Jefferies, W. Mazur, J. Glowacki, J. Hu, M. Lazar, R.A. Gibbs, J. Liczko, J. Klys, E. Venner, D.M. Muzny, J. Rycaj, J. Bialkowski, E. Kluczewska, Z. Kalarus, S. Jhangiani, H. Al-Khalidi, T. Kukulski, J.R. Lupski, W.J. Craigen, and M.N. Bainbridge. 2017. Novel Genetic Triggers and Genotype-Phenotype Correlations in Patients With Left Ventricular Noncompaction. *Circ Cardiovasc Genet*. 10.

Morita, H., H.L. Rehm, A. Menesses, B. McDonough, A.E. Roberts, R. Kucherlapati, J.A. Towbin, J.G. Seidman, and C.E. Seidman. 2008. Shared genetic causes of cardiac hypertrophy in children and adults. *N Engl J Med*. 358:1899-1908.

Nakashima, Y., T. Kubo, K. Sugiura, Y. Ochi, A. Takahashi, Y. Baba, T. Hirota, N. Yamasaki, A. Kimura, Y.L. Doi, and H. Kitaoka. 2020. Lifelong Clinical Impact of the Presence of Sarcomere Gene Mutation in Japanese Patients With Hypertrophic Cardiomyopathy. *Circ J*. 84:1846-1853.

Nguyen, T.V., M.T. Tran Vu, T.N.P. Do, T.H.N. Tran, T.H. Do, T.M.H. Nguyen, B.N. Tran Huynh, L.A. Le, N.T. Nguyen Pham, T.D.A. Nguyen, T.M.N. Nguyen, N.H.P. Le, V. Pham Nguyen, and T.D. Ho Huynh. 2021. Genetic Determinants and Genotype-Phenotype Correlations in Vietnamese Patients With Dilated Cardiomyopathy. *Circ J*. 85:1469-1478.

Norrish, G., J. Jager, E. Field, E. Quinn, H. Fell, E. Lord, M.N. Cicerchia, J.P. Ochoa, E. Cervi, P.M. Elliott, and J.P. Kaski. 2019. Yield of Clinical Screening for Hypertrophic Cardiomyopathy in Child First-Degree Relatives. *Circulation*. 140:184-192.

Olson, T.M., N.Y. Kishimoto, F.G. Whitby, and V.V. Michels. 2001. Mutations that alter the surface charge of alpha-tropomyosin are associated with dilated cardiomyopathy. *J Mol Cell Cardiol*. 33:723-732.

Orzechowski, M., S. Fischer, J.R. Moore, W. Lehman, and G.P. Farman. 2014. Energy landscapes reveal the myopathic effects of tropomyosin mutations. *Arch Biochem Biophys*. 564:89-99.

Otsuka, H., T. Arimura, T. Abe, H. Kawai, Y. Aizawa, T. Kubo, H. Kitaoka, H. Nakamura, K. Nakamura, H. Okamoto, F. Ichida, M. Ayusawa, S. Nunoda, M. Isobe, M. Matsuzaki, Y.L. Doi, K. Fukuda, T. Sasaoka, T. Izumi, N. Ashizawa, and A. Kimura. 2012. Prevalence and distribution of sarcomeric gene mutations in Japanese patients with familial hypertrophic cardiomyopathy. *Circ J*. 76:453-461.

Parrott, A., P.R. Khoury, A.R. Shikany, A. Lorts, C.R. Villa, and E.M. Miller. 2020. Investigation of de novo variation in pediatric cardiomyopathy. *Am J Med Genet C Semin Med Genet*. 184:116-123.

Poloni, G., M. Calore, I. Rigato, E. Marras, G. Minervini, E. Mazzotti, A. Lorenzon, I.E.A. Li Mura, A. Telatin, I. Zara, B. Simionati, M. Perazzolo Marra, J. Ponti, G. Occhi, L. Vitiello, L. Daliento, G. Thiene, C. Basso, D. Corrado, S. Tosatto, B. Bauce, A. Rampazzo, and M. De Bortoli. 2019. A targeted next-generation gene panel reveals a novel heterozygous nonsense variant in the TP63 gene in patients with arrhythmogenic cardiomyopathy. *Heart Rhythm*. 16:773-780.

Probst, S., E. Oechslin, P. Schuler, M. Greutmann, P. Boye, W. Knirsch, F. Berger, L. Thierfelder, R. Jenni, and S. Klaassen. 2011. Sarcomere gene mutations in isolated left ventricular noncompaction cardiomyopathy do not predict clinical phenotype. *Circ Cardiovasc Genet*. 4:367-374.

Pua, C.J., N. Tham, C.W.L. Chin, R. Walsh, C.C. Khor, C.N. Toepfer, G.G. Repetti, A.C. Garfinkel, J.F. Ewoldt, P. Cloonan, C.S. Chen, S.Q. Lim, J. Cai, L.Y. Loo, S.C. Kong, C.W.K. Chiang, N. Whiffin, A. de Marvao, P.M. Lio, A.A. Hii, C.X. Yang, T.T. Le, Y. Bylstra, W.K. Lim, J.X. Teo, K. Padilha, G.V. Silva, B. Pan, R. Govind, R.J. Buchan, P.J.R. Barton, P. Tan, R. Foo, J.W.L. Yip, R.C.C. Wong, W.X. Chan, A.C. Pereira, H.C. Tang, S.S. Jamuar, J.S. Ware, J.G. Seidman, C.E. Seidman, and S.A. Cook. 2020. Genetic Studies of Hypertrophic Cardiomyopathy in Singaporeans Identify Variants in TNNI3 and TNNT2 That Are Common in Chinese Patients. *Circ Genom Precis Med*. 13:424-434.

Pugh, T.J., M.A. Kelly, S. Gowrisankar, E. Hynes, M.A. Seidman, S.M. Baxter, M. Bowser, B. Harrison, D. Aaron, L.M. Mahanta, N.K. Lakdawala, G. McDermott, E.T. White, H.L. Rehm, M. Lebo, and B.H. Funke. 2014. The landscape of genetic variation in dilated cardiomyopathy as surveyed by clinical DNA sequencing. *Genet Med*. 16:601-608.

Quiat, D., L. Witkowski, H. Zouk, K.P. Daly, and A.E. Roberts. 2020. Retrospective Analysis of Clinical Genetic Testing in Pediatric Primary Dilated Cardiomyopathy: Testing Outcomes and the Effects of Variant Reclassification. *J Am Heart Assoc*. 9:e016195.

Racca, A.W., M.J. Rynkiewicz, N. LaFave, A. Ghosh, W. Lehman, and J.R. Moore. 2020. M8R tropomyosin mutation disrupts actin binding and filament regulation: The beginning affects the middle and end. *J Biol Chem*. 295:17128-17137.

Rajan, S., R.P. Ahmed, G. Jagatheesan, N. Petrashevskaya, G.P. Boivin, D. Urboniene, G.M. Arteaga, B.M. Wolska, R.J. Solaro, S.B. Liggett, and D.F. Wieczorek. 2007. Dilated cardiomyopathy mutant tropomyosin mice develop cardiac dysfunction with significantly decreased fractional shortening and myofilament calcium sensitivity. *Circ Res*. 101:205-214.

Regitz-Zagrosek, V., J. Erdmann, E. Wellnhofer, J. Raible, and E. Fleck. 2000. Novel mutation in the alpha-tropomyosin gene and transition from hypertrophic to hypocontractile dilated cardiomyopathy. *Circulation*. 102:E112-116.

Risi, C., J. Eisner, B. Belknap, D.H. Heeley, H.D. White, G.F. Schroder, and V.E. Galkin. 2017. Ca(2+)-induced movement of tropomyosin on native cardiac thin filaments revealed by cryoelectron microscopy. *Proc Natl Acad Sci U S A*. 114:6782-6787.

Robyns, T., J. Breckpot, D. Nuyens, B. Vandenberk, A. Corveleyn, C. Kuiperi, L. Van Aelst, J. Van Cleemput, and R. Willems. 2020. Clinical and ECG variables to predict the outcome of genetic testing in hypertrophic cardiomyopathy. *Eur J Med Genet*. 63:103754.

Ross, S.B., E.S. Singer, E. Driscoll, N. Nowak, L. Yeates, R. Puranik, R.W. Sy, S. Rajagopalan, A. Barratt, J. Ingles, R.D. Bagnall, and C. Semsarian. 2020. Genetic architecture of left ventricular noncompaction in adults. *Hum Genome Var*. 7:33.

Schultze-Berndt, A., J. Kuhnisch, C. Herbst, F. Seidel, N. Al-Wakeel-Marquard, J. Dartsch, S. Theisen, W. Knirsch, R. Jenni, M. Greutmann, E. Oechslin, F. Berger, and S. Klaassen. 2021. Reduced Systolic Function and Not Genetic Variants Determine Outcome in Pediatric and Adult Left Ventricular Noncompaction Cardiomyopathy. *Front Pediatr*. 9:722926.

Seo, G.H., T. Kim, I.H. Choi, J.Y. Park, J. Lee, S. Kim, D.G. Won, A. Oh, Y. Lee, J. Choi, H. Lee, H.G. Kang, H.Y. Cho, M.H. Cho, Y.J. Kim, Y.H. Yoon, B.L. Eun, R.J. Desnick, C. Keum, and B.H. Lee. 2020. Diagnostic yield and clinical utility of whole exome sequencing using an automated variant prioritization system, EVIDENCE. *Clin Genet*. 98:562-570.

Simm, D., K. Hatje, and M. Kollmar. 2015. Waggawagga: comparative visualization of coiled-coil predictions and detection of stable single alpha-helices (SAH domains). *Bioinformatics*. 31:767-769.

Sousa, A., P. Canedo, O. Azevedo, L. Lopes, T. Pinho, M. Baixia, F. Rocha-Goncalves, L. Goncalves, J.S. Cardoso, J.C. Machado, E. Martins, and F. investigators. 2019. Molecular characterization of Portuguese patients with dilated cardiomyopathy. *Rev Port Cardiol (Engl Ed)*. 38:129-139.

Takasaki, A., K. Hirono, Y. Hata, C. Wang, M. Takeda, J.K. Yamashita, B. Chang, H. Nakaoka, M. Okabe, N. Miyao, K. Saito, K. Ibuki, S. Ozawa, M. Sekine, N. Yoshimura, N. Nishida, N.E. Bowles, and F. Ichida. 2018. Sarcomere gene variants act as a genetic trigger underlying the development of left ventricular noncompaction. *Pediatr Res*. 84:733-742.

Thierfelder, L., H. Watkins, C. MacRae, R. Lamas, W. McKenna, H.P. Vosberg, J.G. Seidman, and C.E. Seidman. 1994. Alpha-tropomyosin and cardiac troponin T mutations cause familial hypertrophic cardiomyopathy: a disease of the sarcomere. *Cell*. 77:701-712.

Tian, T., J. Wang, H. Wang, K. Sun, Y. Wang, L. Jia, Y. Zou, R. Hui, X. Zhou, and L. Song. 2015. A low prevalence of sarcomeric gene variants in a Chinese cohort with left ventricular non-compaction. *Heart Vessels*. 30:258-264.

Tran Vu, M.T., T.V. Nguyen, N.V. Huynh, H.T. Nguyen Thai, V. Pham Nguyen, and T.D. Ho Huynh. 2019. Presence of Hypertrophic Cardiomyopathy Related Gene Mutations and Clinical Manifestations in Vietnamese Patients With Hypertrophic Cardiomyopathy. *Circ J*. 83:1908-1916.

van de Meerakker, J.B., I. Christiaans, P. Barnett, R.H. Lekanne Deprez, A. Ilgun, O.R. Mook, M.M. Mannens, J. Lam, A.A. Wilde, A.F. Moorman, and A.V. Postma. 2013. A novel alpha-tropomyosin mutation associates with dilated and non-compaction cardiomyopathy and diminishes actin binding. *Biochim Biophys Acta*. 1833:833-839.

Van Driest, S.L., E.G. Ellsworth, S.R. Ommen, A.J. Tajik, B.J. Gersh, and M.J. Ackerman. 2003. Prevalence and spectrum of thin filament mutations in an outpatient referral population with hypertrophic cardiomyopathy. *Circulation*. 108:445-451.

van Lint, F.H.M., O.R.F. Mook, M. Alders, H. Bikker, R.H. Lekanne Dit Deprez, and I. Christiaans. 2019. Large next-generation sequencing gene panels in genetic heart disease: yield of pathogenic variants and variants of unknown significance. *Neth Heart J*. 27:304-309.

van Spaendonck-Zwarts, K.Y., I.A. van Rijsingen, M.P. van den Berg, R.H. Lekanne Deprez, J.G. Post, A.M. van Mil, F.W. Asselbergs, I. Christiaans, I.M. van Langen, A.A. Wilde, R.A. de Boer, J.D. Jongbloed, Y.M. Pinto, and J.P. van Tintelen. 2013. Genetic analysis in 418 index patients with idiopathic dilated cardiomyopathy: overview of 10 years' experience. *Eur J Heart Fail*. 15:628-636.

Verdonschot, J.A.J., M.R. Hazebroek, I.P.C. Krapels, M. Henkens, A. Raafs, P. Wang, J.J. Merken, G.R.F. Claes, E.K. Vanhoutte, A. van den Wijngaard, S.R.B. Heymans, and H.G. Brunner. 2020. Implications of Genetic Testing in Dilated Cardiomyopathy. *Circ Genom Precis Med*. 13:476-487.

Viswanathan, S.K., H.K. Sanders, J.W. McNamara, A. Jagadeesan, A. Jahangir, A.J. Tajik, and S. Sadayappan. 2017. Hypertrophic cardiomyopathy clinical phenotype is independent of gene mutation and mutation dosage. *PLoS One*. 12:e0187948.

Walsh, R., K.L. Thomson, J.S. Ware, B.H. Funke, J. Woodley, K.J. McGuire, F. Mazzarotto, E. Blair, A. Seller, J.C. Taylor, E.V. Minikel, C. Exome Aggregation, D.G. MacArthur, M. Farrall, S.A. Cook, and H. Watkins. 2017. Reassessment of Mendelian gene pathogenicity using 7,855 cardiomyopathy cases and 60,706 reference samples. *Genet Med*. 19:192-203.

Wang, C., Y. Hata, K. Hirono, A. Takasaki, S.W. Ozawa, H. Nakaoka, K. Saito, N. Miyao, M. Okabe, K. Ibuki, N. Nishida, H. Origasa, X. Yu, N.E. Bowles, F. Ichida, and L.S.C. for. 2017. A Wide and Specific Spectrum of Genetic Variants and Genotype-Phenotype Correlations Revealed by Next-Generation Sequencing in Patients with Left Ventricular Noncompaction. *J Am Heart Assoc*. 6.

Wang, Z., M. Grange, S. Pospich, T. Wagner, A.L. Kho, M. Gautel, and S. Raunser. 2022. Structures from intact myofibrils reveal mechanism of thin filament regulation through nebulin. *Science*. 375:eabn1934.

Wilson, K.D., P. Shen, E. Fung, I. Karakikes, A. Zhang, K. InanlooRahatloo, J. Odegaard, K. Sallam, R.W. Davis, G.K. Lui, E.A. Ashley, C. Scharfe, and J.C. Wu. 2015. A Rapid, High-Quality, Cost-Effective, Comprehensive and Expandable Targeted Next-Generation Sequencing Assay for Inherited Heart Diseases. *Circ Res*. 117:603-611.

Witjas-Paalberends, E.R., N. Piroddi, K. Stam, S.J. van Dijk, V.S. Oliviera, C. Ferrara, B. Scellini, M. Hazebroek, F.J. ten Cate, M. van Slegtenhorst, C. dos Remedios, H.W. Niessen, C. Tesi, G.J. Stienen, S. Heymans, M. Michels, C. Poggesi, and J. van der Velden. 2013. Mutations in MYH7 reduce the force generating capacity of sarcomeres in human familial hypertrophic cardiomyopathy. *Cardiovasc Res*. 99:432-441.

Xiong, H.Y., B. Alipanahi, L.J. Lee, H. Bretschneider, D. Merico, R.K. Yuen, Y. Hua, S. Gueroussov, H.S. Najafabadi, T.R. Hughes, Q. Morris, Y. Barash, A.R. Krainer, N. Jojic, S.W. Scherer, B.J. Blencowe, and B.J. Frey. 2015. RNA splicing. The human splicing code reveals new insights into the genetic determinants of disease. *Science*. 347:1254806.

Yamauchi-Takihara, K., C. Nakajima-Taniguchi, H. Matsui, Y. Fujio, K. Kunisada, S. Nagata, and T. Kishimoto. 1996. Clinical implications of hypertrophic cardiomyopathy associated with mutations in the alpha-tropomyosin gene. *Heart*. 76:63-65.

Yoneda, Z.T., K.C. Anderson, J.A. Quintana, M.J. O'Neill, R.A. Sims, A.M. Glazer, C.M. Shaffer, D.M. Crawford, T. Stricker, F. Ye, Q. Wells, L.W. Stevenson, G.F. Michaud, D. Darbar, S.A. Lubitz, P.T. Ellinor, D.M. Roden, and M.B. Shoemaker. 2021. Early-Onset Atrial Fibrillation and the Prevalence of Rare Variants in Cardiomyopathy and Arrhythmia Genes. *JAMA Cardiol*. 6:1371-1379.

Zimmerman, R.S., S. Cox, N.K. Lakdawala, A. Cirino, D. Mancini-DiNardo, E. Clark, A. Leon, E. Duffy, E. White, S. Baxter, M. Alaamery, L. Farwell, S. Weiss, C.E. Seidman, J.G. Seidman, C.Y. Ho, H.L. Rehm, and B.H. Funke. 2010. A novel custom resequencing array for dilated cardiomyopathy. *Genet Med*. 12:268-278.

Zou, Y., J. Wang, X. Liu, Y. Wang, Y. Chen, K. Sun, S. Gao, C. Zhang, Z. Wang, Y. Zhang, X. Feng, Y. Song, Y. Wu, H. Zhang, L. Jia, H. Wang, D. Wang, C. Yan, M. Lu, X. Zhou, L. Song, and R. Hui. 2013. Multiple gene mutations, not the type of mutation, are the modifier of left ventricle hypertrophy in patients with hypertrophic cardiomyopathy. *Mol Biol Rep*. 40:3969-3976.
